# Insights from a year of field deployments inform the conservation of an endangered estuarine fish

**DOI:** 10.1093/conphys/coae088

**Published:** 2024-12-26

**Authors:** Brittany E Davis, Bruce G Hammock, Nicole Kwan, Catarina Pien, Heather Bell, Rosemary Hartman, Melinda R Baerwald, Brian Schreier, Daphne Gille, Shawn Acuña, Swee Teh, Tien-Chieh Hung, Luke Ellison, Dennis E Cocherell, Nann A Fangue

**Affiliations:** California Department of Water Resources, 3500 Industrial Blvd., West Sacramento, CA 95691, USA; Department of Anatomy, Physiology, and Cell Biology, University of California Davis, 1 Shields Ave., Davis, CA 95616, USA; California Department of Water Resources, 3500 Industrial Blvd., West Sacramento, CA 95691, USA; California Department of Water Resources, 3500 Industrial Blvd., West Sacramento, CA 95691, USA; U.S. Bureau of Reclamation Bay-Delta Office, 801 I St., Suite 140, Sacramento, CA 95814, USA; Department of Wildlife, Fish, and Conservation Biology, University of California Davis, 1 Shields Ave., Davis, CA 95616, USA; California Department of Water Resources, 3500 Industrial Blvd., West Sacramento, CA 95691, USA; California Department of Water Resources, 3500 Industrial Blvd., West Sacramento, CA 95691, USA; California Department of Water Resources, 3500 Industrial Blvd., West Sacramento, CA 95691, USA; California Department of Water Resources, 3500 Industrial Blvd., West Sacramento, CA 95691, USA; Metropolitan Water District of Southern California, 1121 L St., Suite 900, Sacramento, CA 95814, USA; Department of Anatomy, Physiology, and Cell Biology, University of California Davis, 1 Shields Ave., Davis, CA 95616, USA; Fish Conservation and Culture Laboratory, Department of Biological and Agricultural Engineering, University of California Davis, 1 Shields Ave., Davis, CA 95616, USA; Fish Conservation and Culture Laboratory, Department of Biological and Agricultural Engineering, University of California Davis, 1 Shields Ave., Davis, CA 95616, USA; U.S. Bureau of Reclamation Bay-Delta Office, 801 I St., Suite 140, Sacramento, CA 95814, USA; U.S. Bureau of Reclamation Bay-Delta Office, 801 I St., Suite 140, Sacramento, CA 95814, USA

**Keywords:** Cage, delta smelt, habitat, physiology, population recovery, season, survival, temperature, thermal tolerance, wild, zooplankton

## Abstract

Freshwater fishes are increasingly facing extinction. Some species will require conservation intervention such as habitat restoration and/or population supplementation through mass-release of hatchery fish. In California, USA, a number of conservation strategies are underway to increase abundance of the endangered Delta Smelt (*Hypomesus transpacificus*); however, it is unclear how different estuarine conditions influence hatchery fish. The goal of this study was to evaluate a year of Delta Smelt field deployments to inform species conservation strategies of suitable conditions for smelt physiology. Hatchery-reared Delta Smelt was deployed in experimental cages (seven deployments) throughout the Estuary in the winter, summer and fall of 2019. Effects of season and location of cage deployments on fish health (condition factor and histological condition of liver and gill), growth, thermal tolerance and survival were evaluated. The results indicate both seasonal and location differences, with high survival in the winter (100%) and fall (88–92%) compared to lower survival in summer (67%). In the summer, one of the study sites had no surviving fish following high temperature exposure, which peaked ~26°C. After 29 days in the cages, surviving Delta Smelt in summer and fall showed signs of nutritional stress that may be related to biofouling of the cages limiting passive food inputs, restriction of natural foraging behaviour by containment in the cages, and water temperatures that were too high given the chronically low pelagic productivity in the Estuary overall. Field measurements of upper thermal tolerance (CTmax) following caging exposures suggest that laboratory measures of CTmax may overestimate the realized tolerance in a more stochastic field environment. This study demonstrates the utility of using cages as an experimental tool to better understand aspects of Delta Smelt physiological responses to environmental changes across estuarine habitats in a more natural-field setting, while also highlighting potential limitations of using cages.

## Introduction

Freshwater fish in North America are becoming extinct at three times the global rate (Pimm *et al.,* 2014). California fish species are particularly imperilled, as 5% of its native inland fishes are extinct and 78% are declining (Moyle *et al.,* 2011). Conservation actions to support imperilled California fishes include direct restoration of habitat and/or indirect enhancements of key habitat attributes (e.g. access to historical habitat, food, improved water quality and passage) (Sommer, 2020; Sommer et al., 2020; Serra-Llobet *et al.,* 2022). For example, a levee was moved away from the Consumnes River, California, reuniting the river with a portion of its historical floodplain, thereby increasing riparian vegetation and habitat for floodplain dependent species (Serra-Llobet *et al.,* 2022). In a second example, a channelized reach of the Merced River that was dredged for gold was restored with a mixture of gravels and cobbles, resulting in increased Chinook Salmon (*Oncorhynchus tshawytscha*) spawning and use during both droughts and floods (Brown *et al.,* 2022). However, similar efforts may be insufficient or infeasible to support population resiliency for some species such as California native and endangered Delta Smelt (*Hypomesus transpacificus*), making conservation measures such as reintroduction, translocation and population supplementation necessary. In cases where a species is already rare or endangered, hatcheries may maintain a refugial population and those captive bred fish can be used to supplement the wild population (Johnson and Jensen, 1991). However, questions arise about how hatchery-reared fish will transition to stochastic environmental conditions in the wild, including whether the released fish will feed, grow, survive and reproduce.

It can be challenging to evaluate the effectiveness of habitat restoration and supplementation actions meant to benefit a rare species (Diefenderfer *et al.,* 2021) because of low sample sizes. Field surveys, the commonly applied approach, may only have a handful of observations of rare fish to observe the impact of habitat restoration actions. However, deploying hatchery populations in controlled but natural settings using enclosures, can provide a first step towards managing supplementation and habitat actions because it allows a ‘bioassay’ to measure fish responses before actions occur. Enclosures can also be important tools for studying life-history, contaminant exposure and to inform adaptive management of species. For example, enclosures were used to identify limiting factors of endangered Razorback Suckers (*Xyrauchen texanus*) and Bonytail (*Gila elegans*) on the Green River, Utah (Christopherson *et al.,* 2004). Similarly, cages have been used to test morphological variation in hatchery-reared, endangered June Suckers (*Chasmistes liorus*) in a more natural environment in Provo Bay of Utah Lake (Belk *et al.,* 2008). Aha *et al.* (2021) used cages to compare habitats for rearing juvenile Chinook Salmon in Suisun Marsh (SM), California, while Lusardi *et al.* (2020) examined relationships among growth, water temperature and prey availability of Coho Salmon (*Oncorhynchus kisutch*) caged along a spring-fed river in the Shasta River basin. Enclosures can even be used as an alternative to hatcheries for breeding endangered fishes (Billman and Belk, 2009), and may be a useful tool for ‘soft-release’ of supplementation populations into the environment to increase postrelease survival (Tetzlaff *et al.,* 2019; Baerwald *et al.,* 2023). Thus, cages offer a compromise between the controlled conditions of laboratory experiments and the more realistic but uncontrolled conditions of natural experiments (*sensu*  Diamond, 1983).

The Delta Smelt is a small, pelagic, euryhaline osmerid that is endemic to the Sacramento-San Joaquin Delta and San Francisco Bay Estuary, California (referred to as ‘the Estuary’ hereafter). Delta Smelt were once one of the most abundant pelagic fishes in the Estuary but have declined steeply since the 1970’s, with a population crash after 2011, and are now exceedingly rare in the wild (Bennett, 2005; Teh *et al.,* 2020; Hung *et al.,* 2022). This decline is thought to have resulted from a combination of anthropogenic stressors (e.g. habitat loss, water exports, food limitation, invasive species, reviewed in Yanagitsuru *et al.,* 2022). Delta Smelt was listed under the federal and state Endangered Species Act (ESA) as threatened in 1993 (USFWS, 1993) and uplisted to endangered on the California ESA in 2009 (CDFW 2023). Following its ESA-listing, a captive breeding program for the species began, with a founding population of wild-caught Delta Smelt in 2008 (Lindberg *et al.,* 2013) resulting in nearly 15 years of a genetically managed refuge population. In addition to hatchery conservation efforts, state and federal resource managers have also adaptively managed a variety of habitat actions (Sommer, 2020) to improve suitable habitat conditions and resiliency for Delta Smelt (Jassby *et al.,* 1995; Nobriga *et al.,* 2008; Hobbs *et al.,* 2019; Yanagitsuru *et al.,* 2022). These actions include: tidal operation of a salinity control gate to freshen SM (Sommer *et al.,* 2020), increased freshwater flows to shift the salinity field seaward (Feyrer *et al.,* 2011; Kimmerer *et al.,* 2013; Hassrick *et al.,* 2023) and redirecting Sacramento River water or agriculture drainage into the Yolo Bypass (YB) to subsize plankton in the lower Sacramento River (Frantzich *et al.,* 2021). A few actions have met the intended habitat objectives (e.g. increasing low salinity habitat, and overlap with more turbid and cooler habitat), while others have been variable (e.g. subsizing zooplankton; Stillway *et al.,* 2024), and none have resulted in a measureable population response.

Given the scarcity of Delta Smelt in the wild, it is nearly impossible to evaluate the efficacy of these management actions using specimens collected during fish monitoring and survey efforts. Therefore in 2019, *in situ* enclosures for hatchery Delta Smelt were designed and deployed to ask whether hatchery Delta Smelt could transition to and survive in the wild and further, how habitat actions may benefit Delta Smelt (Baerwald *et al.,* 2023). Enclosures were carefully tested and constructed around the unique physiological and behavioural requirements of Delta Smelt (Gille *et al.,* 2024), and the first deployment study (Baerwald *et al.,* 2023) occurred as supplementation of the wild Delta Smelt population with hatchery-raised Delta Smelt was being considered. Questions regarding how to best release fish into the wild quickly emerged, including when, where and how fish should be introduced and acclimatized in cages before release.

The high survival of hatchery-reared Delta Smelt reported in the initial enclosure study (Baerwald *et al.,* 2023) paved the way for the use of enclosures as a tool to evaluate management actions but also provided an opportunity to field-acclimatize smelt in enclosures to assess key fitness-related traits such as growth, reproduction, survival and tolerance (Boisclair and Sirois, 1993; Johnsson *et al.,* 2000; Fangue and Bennett, 2003). Previous studies of thermal physiology of marine fishes suggest a difference between laboratory acclimation and field acclimatization, but effects may differ depending on the variability of the habitat or be species specific (Fangue and Bennett, 2003; Fangue *et al.,* 2011). While environmental variables such as salinity and turbidity influence Delta Smelt distributions in the Estuary (Sommer and Mejia, 2013), water temperature, as with other fishes (Fry, 1947), is likely the most important factor impacting Delta Smelt physiology, behaviour and habitat use. Field catch and laboratory acclimation studies indicate Delta Smelt are particularly sensitive to temperature, with sublethal stress and reduced growth demonstrated above 20°C, food consumption limitations above 22°C and limited survival above 25°C (Sommer and Mejia, 2013; Komoroske *et al.,* 2014; Jeffries *et al.,* 2016; Davis *et al.,* 2019a,b; Lewis *et al.,* 2021; Smith and Nobriga, 2023). Continued increases in water temperature in the Estuary will challenge Delta Smelt persistence, regardless of supplementation efforts, by compression of suitable thermal habitat (particularly in summer and fall) (Brown *et al.,* 2016; Bashevkin *et al.,* 2022a; Mahardja *et al.,* 2022). The ability to collect field-acclimatized information for Delta Smelt, which may differ from laboratory-derived data in important ways, may influence current assessments of where, for example, thermally suitable habitat for field deployed Delta Smelt or supplementation releases occur.

The overall goal of this study was to integrate and evaluate a year of Delta Smelt deployments to determine if temporal and spatial effects of habitat conditions in the Estuary impact Delta Smelt physiology. Additionally, provided the opportunity, we also compared thermal tolerance of field-acclimatized Delta Smelt to historical laboratory data of Delta Smelt to inform application of thermal physiology to management of the species. We investigated the following questions: (i) Does the season or location of enclosure deployments influence Delta Smelt physiology (e.g. health including gill and liver condition, growth and survival)? (ii) Does the upper temperature tolerance of caged Delta Smelt vary across caging locations? (iii) Are field measures of temperature tolerance congruent with previous laboratory studies? These study questions were evaluated using seven cage deployments throughout the winter, summer and fall of 2019 at four sites throughout the range of Delta Smelt. We predicted that Delta Smelt would show signs of compromised health and lower survival in summer compared to other seasons due heat stress and reduced food availability, and that fish condition would vary across locations in a single season due to differences in abiotic habitat conditions. For example, we predicted fish physiology metrics would be better in the marsh compared to the river due to overlapping habitat features (e.g. turbid and cooler; Lewis *et al.,* 2021). Finally, we predicted lower field- versus laboratory-derived thermal tolerance measures due to increased variability of abiotic and biotic factors in the field that may require shifts in stress mechanisms and energy allocation.

## Materials and Methods

### Study area and species

**Figure 1:**
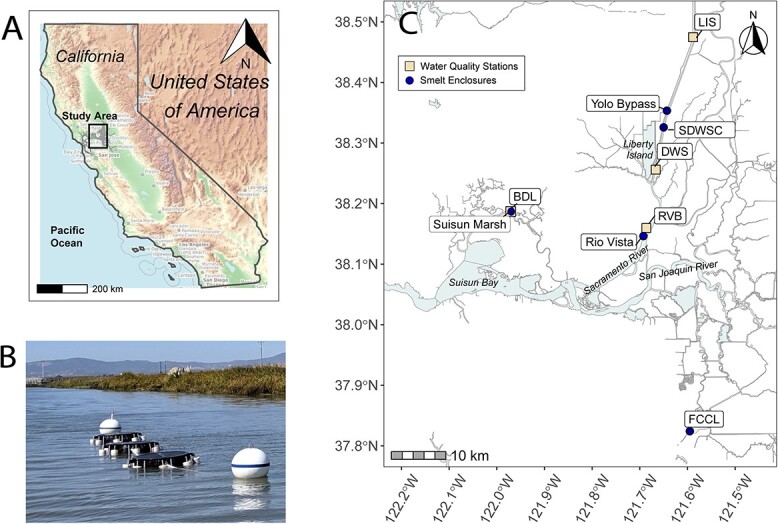
Map (**A**) showing the study area of the San Francisco Estuary, California, USA, (**B**) a picture of Delta Smelt enclosure replicates and a map (**C**) of enclosure deployment sites. Map labels include enclosure sites (blue circles) and locations of continuous water quality stations (yellow squares). SDWSC is the Sacramento River Deepwater Ship Channel and FCCL is the Fish Conservation and Culture Laboratory. Continuous water quality stations are as follows: RV Bridge (RVB), Belden’s Landing (BDL), Deepwater Ship channel (DWS) and Lisbon Weir (LIS). Photograph provided by DWR.

The San Francisco Estuary, California, on the eastern coast of the Pacific Ocean is comprised of the saline San Francisco Bay, Suisun Bay and Marsh and the Delta complex made by the tidal freshwater confluence of the Sacramento and San Joaquin Rivers (Fig. 1). Euryhaline Delta Smelt complete their annual life cycle primarily occupying open water habitats in Suisun Bay, Suisun Marsh, and the Delta. Three life-history phenotypes of Delta Smelt exist, including migratory, freshwater and brackish water residents (Hobbs *et al.,* 2019). The historically dominant migratory phenotype entails adults spawning in spring in freshwater habitat (January-May), after which larvae volitionally move or are advected out of the Delta (March-July) where juveniles rear in brackish water habitat in summer–fall until they are subadults (July-December) (Mahardja *et al.,* 2022). Maturing subadults will migrate back upstream towards freshwater habitat in winter. The dominant migratory phenotype overlaps with seasonal changes that occur in the Estuary, with particularly warm summers where smelt must seek cooler conditions towards brackish water habitat (e.g. Suisun Marsh and Bay), but also find food, which starts to decline in late summer and fall and is typically higher in freshwater compared to more saline habitats (Smith and Nobriga, 2023).

Four study sites were selected throughout the recent range of Delta Smelt: Rio Vista (RV), Sacramento River Deepwater Ship Channel (SDWSC), Yolo Bypass (YB) and Suisun Marsh (SM) (Fig. 1). These sites represent different habitat types and routine monitoring surveys have captured Delta Smelt at all sites in the last ten years (2013–2023) (Bashevkin *et al.,* 2022b; USFWS *et al.,* 2023). The RV site is on the lower main stem of the Sacramento River, adjacent to the RV Army Base. The location is used by migratory Delta Smelt to reach freshwater spawning habitat in the winter/spring or brackish water rearing habitat in the summer (Moyle *et al.,* 2016; Hobbs *et al.,* 2019). The SDWSC is a freshwater, constructed channel that provides ships access to the Port of West Sacramento. It acts as a dead-end slough and is the primary location where Delta Smelt were found in the wild across multiple seasons in recent years (Merz *et al.,* 2011; Davis *et al.,* 2022; Mahardja *et al.,* 2022). The 61 km-long, 24 000 ha engineered YB is a major floodplain of the Sacramento River basin where Delta Smelt were historically detected year-round in its perennial eastern canal (Sommer *et al.,* 2001; Moyle *et al.,* 2016; Mahardja *et al.,* 2019; Stompe *et al.,* 2020), however, the last detection of Delta Smelt was in 2017 (IEP *et al.,* 2022) and water temperature is often unsuitable for smelt survival in the summer. SM comprises approximately 34 000 hectares of tidal marsh, managed wetlands and waterways in southern Solano County. It is the largest remaining wetland near San Francisco Bay and includes more than ten percent of Calfornia’s total remaining wetland area. SM is a wildlife habitat of nationwide importance (SM Preservation Act of 1977), and is considered relatively high-quality habitat for Delta Smelt when salinity is appropriate (e.g. <6 PSU, Hammock *et al.,* 2015; Moyle *et al.,* 2016). The University of California Davis Fish Conservation and Culture Laboratory (FCCL) in Byron, CA was also used as the hatchery reference site, where a captive refuge population of Delta Smelt has been maintained since 2008 (Lindberg *et al.,* 2013).

**Table 1:** Enclosure deployments in 2019

**Location**	**Metric**	**Winter**	**Summer**	**Fall**
RV	Date	Jan 23–Feb 25	Jul 30–Aug 28	Oct 9–Nov 6
	DPH	243–276	194–223	264–292
	n-fish	136	180	180
	n-cage	3	3	3
	% Survival	100	67	89
SDWSC	Date	Feb 27–Mar 27	No enclosures	No enclosures
	DPH	278–306		
	n-fish	120		
	n-cage	3		
	% Survival	98		
SM	Date	No enclosures	No enclosures	Oct 9–Nov 7
	DPH			264–293
	n-fish			180
	n-cage			3
	% Survival			88
YB	Date	No enclosures	Jul 30–Aug 19	Oct 9–Nov 7
	DPH		194	264–293
	n-fish		60	180
	n-cage		1	3
	% Survival		0	92
FCCL	Date	Jan 23–Feb 25 (1), Feb 27–Mar 27 (2)	Jul 29–Aug 29	Oct 8–Nov 5
	DPH	243–276 (1), 278–306 (2)	194–225	263–291
	n	60 (1), 60 (2)	90	90
	% Survival	83 (1), 85 (2)	98	99

Age (days-posthatch, DPH) of fish when put in enclosures and sampled, number of fish deployed (n-fish) and number of enclosure replicates (n-cage) and estimated percentage survival of Delta Smelt are summarized. RV is Rio Vista in the Sacramento River, SDWSC is Sacramento River Deepwater Ship Channel, SM is Suisun Marsh and YB is the Yolo Bypass

#### Deployment of site and season

**Figure 2:**
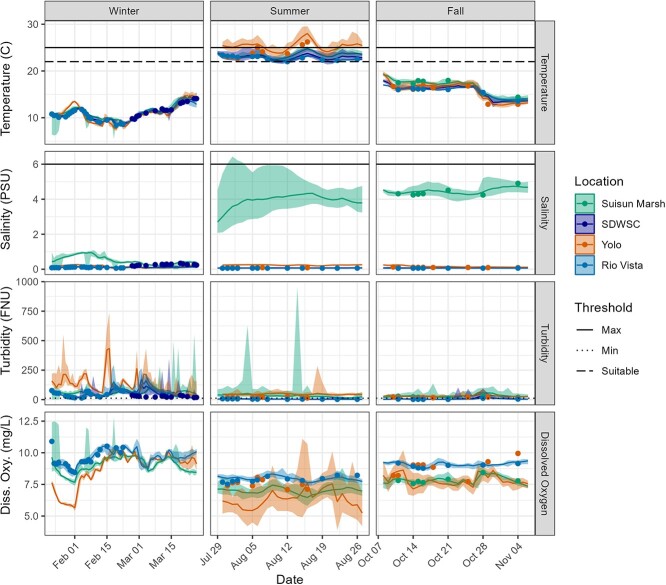
Water quality parameters measured every 15 minutes across seasons and enclosure locations in 2019. Lines indicate daily mean values, bottom and top of coloured ribbon are the daily minimum and maximum values. Overlaid points are discrete water quality data taken weekly at the enclosures and black lines represent Delta Smelt thresholds. The solid black line indicates the maximum temperature Delta Smelt can survive, the dashed line indicates the temperature below which smelt can grow and the dotted line indicates the minimum turbidity (>12 FNU) above which Delta Smelt are normally found.

There were three deployment periods in 2019, winter/spring (January to March, hereafter winter), summer (July to August) and fall (October to November). In winter, enclosures were deployed at RV and at SDWSC as in Baerwald *et al.* (2023), in summer they were deployed at RV and YB and fall at RV, YB and SM (Table 1). The winter deployment period was included given favourable water temperatures for Delta Smelt (range of 8–12°C, Table 2; Fig. 2) and to correlate with the timing of future experimental releases for supplementation of Delta Smelt to the wild. Enclosure deployments were used in the summer and fall to support (i) the future evaluation of habitat and food management actions (e.g. overlap of suitable key physical attributes for Delta Smelt; salinity < 6 PSU, turbidity > 12 FNU and water temperature < 22–25°C [Sommer and Mejia, 2013; Smith and Nobriga, 2023]), (ii) potential efficacy of summer–fall supplementation releases and (iii) test enclosure effects and temperature tolerance of Delta Smelt following field acclimation (range of 15–26°C, Table 2; Fig. 2).

**Table 2:** Discrete water quality measures taken at enclosure checks during the deployments

**Site and season**		**Water temperature (°C)**	**Specific conductivity (μS/cm)**	**Turbidity (FNU)**	**Dissolved oxygen (mg/L)**
RV Winter	Min	8.4	127.0	13.9	8.5
	Mean	10.1	214	46.6	9.5
	Max	11.8	283.0	99.6	10.9
SDWSC Winter	Min	9.7	366.0	17.6	9.6
	Mean	11.9	528.1	27.1	10.3
	Max	14.1	695.0	38.2	10.6
RV Summer	Min	22.0	125.0	2.6	7.5
	Mean	22.8	131.1	4.9	7.9
	Max	23.4	137.2	7.9	8.2
YB Summer	Min	22.6	184.0	14.3	7.1
	Mean	24.1	191.8	19	7.5
	Max	26.2	201.0	26.6	8.1
RV Fall	Min	13.5	120.1	2.5	8.8
	Mean	15.6	130.4	4.8	9
	Max	16.2	134.8	12.3	9.2
YB Fall	Min	12.9	190.0	13	7.7
	Mean	15.7	226.9	17.3	8.8
	Max	16.9	272.0	22.0	10
SM Fall	Min	14.4	7683.0	12.3	7.6
	Mean	16.9	7955.8	21.6	7.8
	Max	18	8802.1	33.0	8.4

The minimum, mean and maximum values are summarized for each deployment location and season. RV is Rio Vista in the Sacramento River, SDWSC is Sacramento River Deepwater Ship Channel, SM is Suisun Marsh and YB is the Yolo Bypass. Discrete measures are overlayed with continuous sonde data in Fig. 2

#### Enclosure design

Each enclosure was designed, built and anchored as described in Baerwald *et al.* (2023) allowing access to pelagic prey and exposure to ambient environmental conditions while preventing Delta Smelt escape. Three replicate enclosures (1.22 m in height and 0.95 m in diameter) were deployed at each site except for the summer at YB (Table 1). The winter and summer deployments used enclosures made of steel mesh painted with black marine-grade paint (Rust-oleum Topside). The fall deployments used enclosures made of aluminium mesh to make them lighter and were powder coated black to improve the surface coat quality. The enclosures were able to move vertically between high and low tide while maintaining the same relative distance between enclosures. At YB, for the summer deployment, only one enclosure was deployed due to fabrication delays of additional enclosures. The summer cage was attached with short cables between two pontoons of a rotary screw trap, which was located centre-channel and not fishing during the period of deployment. There was likely minimal effect of the equipment on cage conditions due to its low draft in the water when not fishing. During the fall deployments the three YB cages were attached and anchored in the same manner as the other sites (and included in statistical comparisons).

#### Fish preparation

Delta Smelt were obtained from the FCCL and acclimated to seasonal water quality conditions and live prey (see below) prior to being transferred to the field in winter, summer and fall. All procedures and experiments abided by animal welfare considerations (IACUC# 21610) and permit regulations (USFWS TE-027742, CDFW CESA MOU# 2081a-2018-0007-R3). Delta Smelt for both winter deployments (RV and SDWSC, as described in Baerwald *et al.,* 2023) were switched from temperature-controlled water (16°C) to unfiltered ambient water (i.e. from the California aqueduct) at 206 days posthatch (dph), from dry pelletized food to live *Artemia* at 207–210 dph and acclimated to higher flows (one day at 15 cm/s and the following days at 31 cm/s) at 223–224 dph. All fish also received Visible Implant Alphanumeric (VIA) tags for individual identification at 207 dph, were weighed and fork length was measured (mean ± std; fork length 5.9 ± 0.5 cm, weight 1.4 ± 0.4 g). About, 384 Delta Smelt were driven to RV on 23 January 2019 (243 dph) and 360 fish were driven to SDWSC on 27 February 2019 (278 dph). See Table 1 for deployment details. For the summer and fall deployments, Delta Smelt from the FCCL underwent a controlled temperature increase from 16 to 18.5°C one week prior to deployments to acclimate them to the warmer temperatures at the field sites. These fish were unable to be VIA tagged due to their smaller size in summer (mean ± std; fork length 4.9 ± 0.4 cm, weight 0.82 ± 0.24 g). Instead, all fish were adipose fin clipped to allow identification of hatchery-origin fish in case of escape. A subsample of 60 fish that were not going into the field were weighed and measured one day prior to field deployment to provide average pre-deployment measurements. On 30 July 2019, 240 Delta Smelt were transported to RV (3 enclosures) and YB (1 enclosure; 194 dph). On 9 October 2019, 540 fish were transported to RV, YB and SM (3 enclosures each site; 264 dph; Table 1).

All fish were transported from FCCL to field sites in insulated, black 19 L buckets with screw-top lids at a density of 34 (RV winter; 1.8 fish/L) and 30 (SDWSC, RV, YB, SM summer/fall; 1.6 fish/L) fish per bucket (Baerwald *et al.,* 2023). The buckets were filled with water at the FCCL, which was raised to 5 PSU salinity using Instant Ocean and saturated with oxygen to decrease transport stress. Upon arrival at each site, buckets were loaded onto a boat and driven to the enclosures. Two buckets of fish were emptied into each enclosure (68 fish/enclosure at RV winter and 60 fish/enclosure at SDWSC, RV YB, SM) using a water-to-water transfer after providing one minute of water exchange between each bucket and the ambient water within each enclosure. These densities were selected to balance food availability with sufficient density to allow for normal shoaling behaviour (Baerwald *et al.,* 2023).

### Field monitoring

During winter the enclosures were checked each weekday. Due to transport and temperature mortality concerns, for the summer and fall deployments enclosures were checked daily during the first week and then weekly thereafter. During each check of the enclosures, the water surface was examined for mortalities (for survival analysis). Mortalities were measured for length and weight (when possible) and stored on ice prior to transferring to a −20°C freezer. Mortalities with degraded bodies were not measured. Water quality and within cage water velocity were measured, and zooplankton were collected adjacent to the enclosures.

#### Water quality

During each enclosure check, a YSI Pro DSS was used to collect discrete measurements of water temperature (°C), dissolved oxygen (DO, mg/L), specific conductivity (μSiemens/cm), pH and turbidity (FNU). In addition to discrete measures, continuous water quality data were collected from multiparameter YSI EXO2 sondes that were nearest to the respective enclosure sites (Fig. 1). Sondes recorded water quality data every 15 minutes for the entire year at Beldon’s Landings (BDL) in SM, Sacramento River at RV Bridge (RVB), Lisbon Weir (LIS) in the YB, and Sacramento River Deep Water Ship Channel (SDWSC). Information about continuous sensors from the Department of Water Resources and US Geologic Survey are provided in Supplementary Table S1.

#### Zooplankton

Zooplankton samples were collected twice per week using a SEA-GEAR conical 0.5 × 2 m plankton net with 53-μm mesh with a General Oceanics flowmeter suspended in the mouth (CDFW SCP S-182970002-20 219-001). The net was towed subsurface for two minutes, within 15 m of the enclosures. Occasionally, due to rough water conditions during the Winter deployment at RV, a smaller (0.3 × 1 m, 53-μm mesh) plankton net was used instead and hand-towed 5–6.5 m along a dock 100 m upstream of the enclosures. The difference in tow speed may cause minor differences in zooplankton; however, because these methods both use the same mesh and include volume estimates, and we consider the data sufficiently comparable (Harris *et al.,* 2000). Samples were stored in 1 L wide-mouth Nalgene bottles and preserved with 5% formalin and dyed with Rose Bengal. Samples were sent to BSA Environmental Services, Inc. (Beachwood, Ohio, USA) for enumeration and identification of mesozooplankton and microzooplankton as described in Frantzich *et al.* (2021). Samples were identified to genus for cladocerans, order for harpacticoids and species and life stage for calanoid and cyclopoid copepods. Microzooplankton were also enumerated, including rotifers, barnacles, copepod nauplii, cladocera nauplii, unidentified nauplii and ostracods.

### Fish sampling and physiology

Following each deployment period, all surviving fish were collected from each enclosure and transported in 19-L insulated black buckets to shore (RV, SM, YB sites) or a laboratory (SDWSC site) where they were euthanized for growth measurements, dissections and/or tissue preservation. Each group of fish was euthanized with an overdose of buffered Tricaine Methanesulfonate (MS-222) after which they were weighed (g) and measured (mm fork length) for growth calculations (pre- and postdeployments). The carcasses were preserved in 10% neutral buffered formalin. Formalin preserved bodies of up to 10 fish per enclosure were subsequently dissected in the laboratory, removing the stomach, liver and left gill arches. Livers were weighed with an analytical balance and liver and gills were fixed in buffered formalin for histopathology. Stomachs were preserved in formalin and sent to the Wetland Ecosystem Team laboratory (Seattle, Washington USA) for gut content identification and later diet analysis. Following the summer and fall deployments but before euthanasia, a subsample of 6–9 fish per enclosure (summer) and 15 fish per enclosure (fall) were transported to shore for measures of critical thermal maximum described in the ‘Temperature Tolerance’ section below.

#### Diets

Delta Smelt diets collected from enclosures were assessed across sites and seasons. The total contents of each stomach were weighed (to 0.0001 g), identified to the lowest taxonomic resolution possible given the extent of digestion, and sorted into life stages when the diagnostic characteristics were identifiable.

#### Histopathology

Gill and liver histopathology was performed on subsamples of fish from each cage according to Teh *et al.* (1997). Sample sizes are in Table 3. Briefly, tissues were dehydrated in an ethanol series and embedded in paraffin, sectioned to 3-μm thickness and stained with haematoxylin and eosin. Livers were screened for glycogen depletion (a measure of energy reserves and a proxy for nutritional stress; Hammock *et al.,* 2020), and other lesion markers indicating fish stress such as fatty vacuolar degeneration, single cell necrosis, inflamation, macrophage aggregate, cytoplasmic inclusion bodies and sinusoid congestion as in Teh *et al.* (2020). Gills were screened for gill lamellar aneurysm, ionocyte hyperplasia, mucous cell hyperplasia, epithelia cell hyperplasia/hypertrophy, secondary lamela edema, gill epithelial cell necrosis, fusion and inflamation (Teh *et al.,* 2020). Lesions were scored on a scale of 0–3, where 0 = not present, 1 = mild, 2 = moderate and 3 = severe. The liver and gill alterations and scoring criteria are described in detail in Teh *et al.* (2020). To provide an overall metric of liver condition and a second overall metric for gill condition, liver lesion markers were summed to produce a liver lesion score and gill lesion markers were summed to produce a gill lesion score for each fish (Hammock *et al.,* 2015; Teh *et al.,* 2020). Liver lesion score for an individual was the summation of the liver alterations listed above except glycogen depletion, which was analyzed seperately because it is not a lesion. Gill lesion score was calculated as the summation of the gill alterations listed above (Teh *et al.,* 2020).

**Table 3:** Gill and liver condition across sites and season

		**FCCL**			RV **(*n* = 59)**			YB			SM			**SDWSC (*n* = 60)**		
	**Lesion**	Mild	Mod	Sev	Mild	Mod	Sev	Mild	Mod	Sev	Mild	Mod	Sev	Mild	Mod	Sev
**Winter**	Liver glycogen depletion	No data			9	15	2	No data			No data			2	27	24
	Liver hydropic vacuolar degeneration				2									2	2	
	Liver single cell necrosis				2									8		
	Liver cytoplasmic inclusion bodies				1									19	14	7
	Liver sinusoid congestion													1		
	Liver inflammation/macrophage aggregate													2		
		FCCL (*n* = 18)			RV (*n* = 21)			YB			SM			SDWSC		
**Summer**	Gill cell necrosis							No data			No data			No data		
	Gill ionocyte hyperplasia															
	Gill mucous cell hyperplasia															
	Gill epithelial cell hyperplasia/hypertrophy															
	Gill inflammation		4													
	Liver glycogen depletion	2	2	2		8	13									
	Liver hydropic vacuolar degeneration				1											
	Liver single cell necrosis	2			2											
	Liver cytoplasmic inclusion bodies	1	1		6	4	4									
	Liver inflammation/macrophage aggregate				1											
		FCCL (*n* = 20)			RV (*n* = 45)			YB (*n* = 45)			SM (*n* = 44)			SDWSC		
**Fall**	Gill cell necrosis				1			6	4					No data		
	Gill ionocyte hyperplasia					10			2							
	Gill mucous cell hyperplasia				4			7								
	Gill epithelial cell hyperplasia/hypertrophy							2			1					
	Gill inflammation				3						1					
	Liver glycogen depletion	1				5	40	1		44			44			
	Liver hydropic vacuolar degeneration				10			3					7			
	Liver single cell necrosis				1		1				1	1	7			
	Liver cytoplasmic inclusion bodies				29	11	1	27	6		30	5	3			
	Liver inflammation/macrophage aggregate				4			4			2	1	10			

Note that blanks in fields indicate zero (not observed). The count of fish observed with Mild, Moderate (Mod) and Severe (Sev) is summarized. Gills were not collected in Winter. RV gills were *n* = 11 in summer (10 were autolyzed). FCCL fish were sampled before (*n* = 40) and after (*n* = 38) for summer and fall deployments. Subsets of the ‘after’ fish were examined histologically (*n* = 18 and 20 for summer and fall)

In addition to the caged and FCCL control fish processed for histology, 20 ‘before deployment’ fish were collected prior to the summer and fall deployments (40 overall) to ensure that the population of FCCL fish used to stock the cages was healthy prior to stocking the cages. Of the 40 fish, one fish had a liver with mild glycogen depletion and one fish had a liver with mild liver inflammation. The livers of the other 38 fish had no lesions. The gills of the ‘before deployment’ fish were also in good condition, with a mean gill lesion score of 0.48 or about one mild gill lesion for every-other fish. These lesions were mainly mild inflammation or mild epithelial cell hyperplasia/hypertrophy. Thus, the histology of the ‘before deployment’ fish indicates that the population of FCCL fish used to load the cages was healthy.

#### Temperature tolerance

Upper temperature tolerance was determined for Delta Smelt after 4 weeks of field acclimatization in the enclosures using critical thermal methodology (CTmax; Beitinger *et al.,* 2000), with modifications for field application. CTmaxima were conducted on a subset of Delta Smelt individuals from RV (*n* = 9 fish) during summer and RV (*n* = 10 fish) and SM (*n* = 10 fish) during fall. Each fish was removed from the field enclosure and placed into a separate 1.25-L chamber, contained in a water bath, at the water temperature of each location (summer RV = 21.0°C, fall RV = 14.2°C, fall SM = 13.5°C) for a 30-minute acclimation period. After 30 minutes, the water bath was heated by two 800 W titanium heat bars, at a rate of 0.3°C per minute, following recommendations by Becker and Genoway (1979). After a loss of equilibrium, fish were quickly removed from their chambers and placed in recovery containers at site temperature; the temperature at which the loss of equilibrium was observed was recorded as the CTmax. In previous thermal tolerance studies of Delta Smelt, fish were given a 24-hour period to recover (Komoroske *et al.,* 2014; Davis *et al.,* 2019a); however, this study was done outside of a laboratory, and therefore, due to in-field constraints the recovery period was reduced to 4 hours. A handling control (*n* = 5 fish) was conducted during the fall experiments at RV and SM to ensure that post-CTmax mortalities were due to high water temperature and not to handling. During the summer RV experiment, one fish was injured prior to acclimation and was not included in the final data set (*n* = 8 fish).

To gain a better understanding of the thermal tolerance of Delta Smelt and to compare our work to previous CTmax laboratory studies, a comparative analysis of the upper thermal tolerance and acclimation potential (i.e. the rate at which fish can acquire thermal tolerance), of laboratory and field studies was completed. All known CTmax data for juvenile Delta Smelt was compiled into a single dataset and CTmax methodology was similar in all the studies. This data set included data from Swanson *et al.* (2000) on wild-caught Delta Smelt, data from hatchery reared smelt collected in the laboratory (Komoroske *et al.* (2014) and Davis *et al.* (2019a)) and the present study using field acclimatized fish exposed to natural variation in temperature and other factors such as turbidity and salinity.

### Statistical analysis

Survival is reported as the percentage of live fish collected at the end of each deployment out of the number introduced to each enclosure. We used *t*-tests to assess whether weight, fork length and condition factor (K) varied between predeployment Delta Smelt at FCCL and postdeployment fish at summer and fall sites. Replicated winter data (RV and DWSC) were assessed in Baerwald *et al.* (2023) and descriptive statistics are provided in the Supplementary Materials (Supplementary Table S2 and Supplementary Fig. S1). We analyzed the effect of season (e.g. winter, summer, fall at RV) and site (within fall) with a random effect of enclosure on each individual’s proportional change in weight (i.e. deltaWeight) and condition factor (i.e. deltaK) using a mixed effects model (*lmerTest* package; Kuznetsova *et al.,* 2017). We compared pairwise differences between seasons and sites using the ‘*emmeans’* package and adjusted the alpha value using the Bonferroni correction (Lenth *et al.,* 2022). Because methodologies of pre-and-post deployment growth measures varied in winter compared to summer and fall (i.e. predeployment batch sampling versus individual tracking), we standardized the datasets by calculating pre-deployment averages for weight and K, then subtracted the averages from each individual’s postdeployment weight and K. To standardize the winter data acquired from Baerwald *et al.* (2023), we filtered the data to three enclosures to match the number of enclosures and cage size in summer and fall (current study) and calculated deltaWeight and deltaK. Baerwald *et al.* (2023) found small and large cage effects to be the same in winter deployments, which led to the use of small cages in subsequent enclosure studies.

Zooplankton and diet data were simplified to major taxonomic groups (amphipods, calanoid copepods, cyclopoid copepods, harpacticoid copepods, cladocera, Diptera and ‘other’). For each sample, abundance of each taxonomic group was divided by total catch in the sample to calculate relative abundance. To assess the difference in zooplankton communities available for fish consumption across time and space, we used a permutational multivariate analysis of variance (PERMANOVA) on the relative abundance of each major group of zooplankton in zooplankton tows across sites during the fall and across seasons at RV. To assess whether fish were consuming zooplankton in similar proportions to their environment, we performed two additional PERMANOVAs: one modelling community composition versus data type (diet versus zooplankton tow), season and the interaction of datatype and season at RV; the final PERMANOVA modelled community composition versus data type, location and the interaction of data type and location during the fall. Empty stomachs were removed from analysis to meet the requirements of the test. Analyses were performed using R version 4.2.2 with the ‘*adonis*’ package from the ‘*vegan*’ package (Oksanen *et al.,* 2020).

Liver lesions, gill lesions and glycogen depletion were each analyzed with Kruskal-Wallis tests. To test the effect of season we compared five categories: RV during winter, summer and fall and FCCL controls during summer and fall. To test the effect of site on lesion scores and glycogen we compared four locations (RV, SM, Yolo and FCCL control) in fall only. Thus there were six Kruskal–Wallis tests overall. Significant Kruskal–Wallis tests were followed by Steel-Dwass tests to compare each of the categories to one-another. Analyses were performed in JMP Pro version 17.0.

The CTmax of fish caged at RV and SM in fall were compared with a t-test. CTmax of RV fish during summer was not included in a test of seasonal effects due to limited fish survival following the recovery period. We used linear regression to test the effect of acclimation temperature (T_acc)_ and study location (Loc; i.e. compare the field versus lab) on CTmax using the formula: CTmax ~ T_acc_ + Loc. We estimated the acclimation rate of average CTmax data for Delta Smelt with CTmax as a function of acclimation temperature. We acknowledge the limitations of using mean CTmax values; however, individual data were not available for the previous studies.

## Results

### Field monitoring

Water temperature was consistent with California’s Mediterranean climate: cooler temperatures in winter and fall and the warmest temperatures in the summer (Table 2, Fig. 2). While RV, SDWSC and SM were generally similar in water temperature regardless of season, the YB in summer was warmer, with an average temperature of 24.1 or 1.5°C higher than RV, and a maximum temperature over 3°C higher than RV. YB also experienced periods of lower dissolved oxygen compared to other sites. Salinity on average was higher in SM compared to other sites (roughly 4 PSU compared to 0 PSU). Turbidity at RV was higher in the winter (average 47 FNU) compared to summer and fall deployment periods (average 5 FNU); however, during fall season turbidity was relatively higher at SM (22 FNU), then YB (17 FNU) and RV (12 FNU).

### Survival and growth

Generally, fish survival varied with season, but was similar across sites within a season, with the exception of summer (Table 1). At RV, the highest survival was observed in the winter season (100%) as compared to fall (89%) and summer (67%). Survival of fish at YB was higher in the fall (92%) compared to 0% survival in summer, when water temperature peaked at 26.2°C (based on discrete water data, Table 2). During winter, survival at RV and SDWSC differed by only ~2%, while in fall survival at RV, SM and YB differed by 3–4%.

Delta Smelt in summer had a significant decrease in condition factor after the deployments at both RV (*t*_(156.7)=_3.36, *P* < 0.001) and FCCL (*t*_(144.8)_ = 3.57, *P* < 0.001) compared to before, with no change in weight of fish at RV or FCCL (*P* > 0.05); however, fork length increased in FCCL fish (*t*_(140.4)_ = −3.86, *P* < 0.001). In fall, Delta Smelt condition factor, weight and fork length decreased after field deployments at all sites (*P* < 0.001), with the exception of fork length of fish at RV, which showed no change in length postdeployment (statistics summaries in Supplementary Table S2, Supplementary Fig. S1).

**Figure 3:**
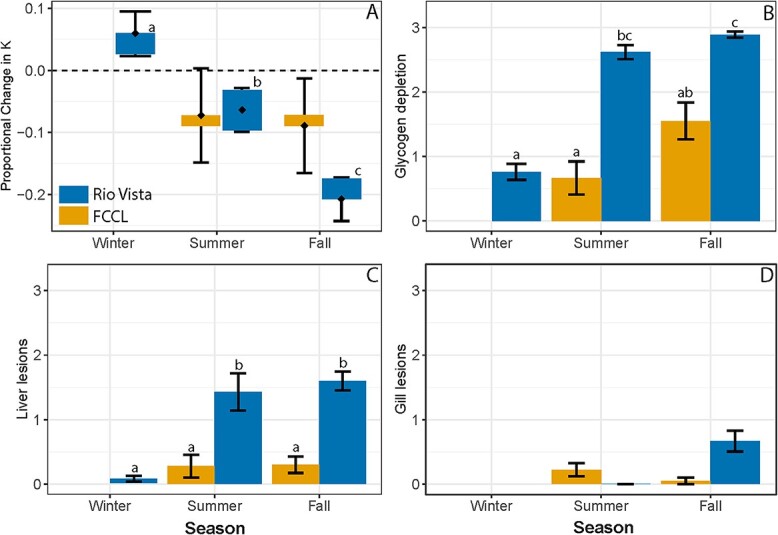
Delta Smelt physiological responses across seasons at RV in Sacramento River in reference to the FCCL control fish (Fish Conservation and Culture Lab). (**A**) Modelled estimate of proportional change in fish condition factor (as K, with 95% confidence intervals), with bars that overlap indicating a lack of significant difference. Mean (±SE) (**B**) liver glycogen depletion score (**C**) liver lesion score and (**D**) gill lesion score. Concurrent FCCL controls were not collected in winter, nor were gills of the fish caged at RV in winter. Different letters represent a significant difference between seasons at RV or with the FCCL reference fish (*P* < 0.05).

**Figure 4:**
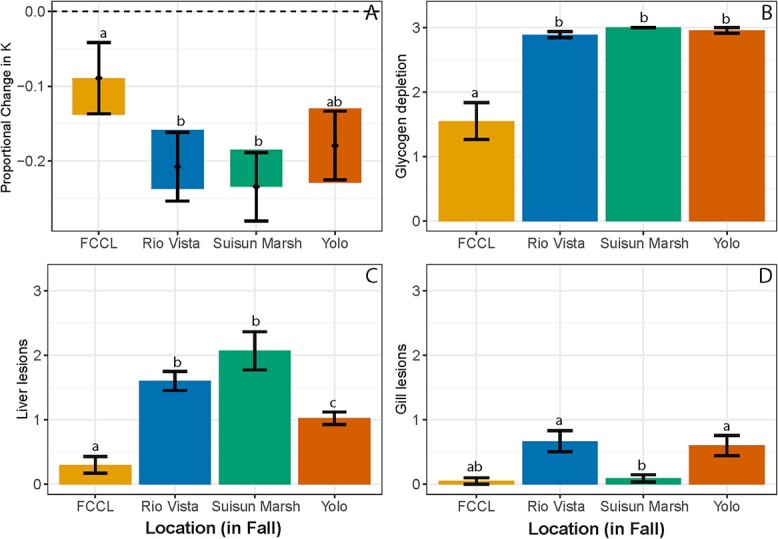
Delta Smelt physiological responses across locations in the fall. (**A**) Modelled estimate of proportional change of fish condition factor (as K, with 95% confidence intervals). Bars that overlap indicate lack of significant difference. Mean (±SE) (**B**) liver glycogen depletion score (**C**) liver lesion score and (**D**) gill lesion score. FCCL is the Fish Conservation and Culture Lab. Different letters represent a significant difference between locations (*P* < 0.05).

Mixed model results of the effects of season (at RV, Fig. 3) and enclosure location (in the fall, Fig. 4) on the change in condition factor and weight (i.e. deltaK and deltaWeight) demonstrate that season and site significantly affected the change in growth of Delta Smelt (*P* < 0.05; model summaries provided in Supplementary Table S3 and S4). At RV the most positive change in condition and weight occurred in the winter season, followed by summer, then fall (Fig. 3A, Supplementary Table S4). In contrast, within the fall season the deltaK of fish differed by enclosure location (Fig. 4A), but deltaWeight was similar (*P* > 0.05) at all fall sites including RV, SM, YB and FCCL (Supplementary Fig. S2). Posthoc comparisons detected differences between reference fish at FCCL to RV and SM. The highest deltaK was at FCCL, followed by YB, whereas the highest deltaWeight was at FCCL followed by RV.

### Zooplankton and diets

**Figure 5:**
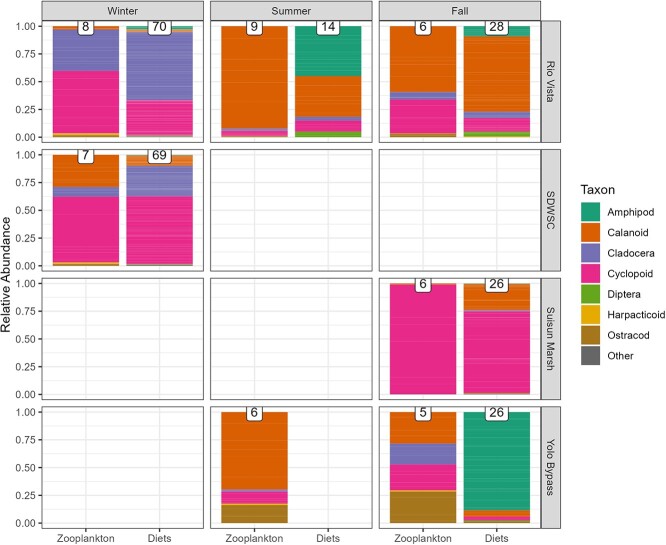
Relative abundance of all zooplankton caught at each site during each deployment season and mean contribution of observed taxa to Delta Smelt diets at each site at the end of each deployment. Microzooplankton (rotifers and nauplii) were removed in order to focus on larger Delta Smelt prey species. To allow for diets of different sized fish to contribute equally, proportion was calculated by taking the abundance of each taxa divided by the total abundance of all taxa per diet and then averaging across individuals from each enclosure. Number of samples is noted above each bar. YB fish in the summer perished and no diets were recovered.

There were major shifts in zooplankton community composition between seasons (*F*_2,22_ = 192.34, *P* = 0.001), with ‘season’ accounting for 95% of the variance in the PERMANOVA model of zooplankton relative abundance by season at RV (Supplementary Table S5). This was driven by higher relative abundance of cladocera in the winter and higher relative abundance of calanoid copepods in the summer (Fig. 5). There were also major differences in zooplankton community composition between site (*F*_2,16_ = 76.06, *P* = 0.001), with ‘site’ accounting for over 90% of the variance in the PERMANOVA model of zooplankton relative abundance by site in the fall (Supplementary Table S5). This was largely driven by more cyclopoid copepods in SM and more ostracods and cladocera in the YB (Fig. 5).

There was a relatively small difference in Delta Smelt diets when compared with zooplankton community composition (Supplementary Table S5). The PERMANOVA model comparing Delta Smelt diets to zooplankton at RV demonstrated roughly 6% of the difference in relative abundance was due to it being a diet versus zooplankton sample (*F*_1,134_ = 15.79, *P* < 0.001), with 40% of the variation due to season (*F*_2,134_ = 50.86, *P* < 0.001). For the model of smelt diet versus zooplankton in the fall, over 50% of the difference in relative abundance was due to location (*F*_2,96_ = 98.29, *P* < 0.001) and only 6% due to it being a diet versus a zooplankton sample (*F*_1,96_ = 23.39, *P* < 0.001). The largest difference between zooplankton and diet samples was the inclusion of Diptera and amphipods in the diet samples, which were absent in zooplankton samples (Fig. 5).

### Histopathology

Glycogen depletion of caged fish varied by the combined influence of season and site (Kruskal-Wallis, χ^2^ = 96.33, P < 0.0001). Mean glycogen depletion was moderate to severe for fish deployed to RV in summer and fall and both were higher than their respective FCCL controls (Steel-Dwass *P* < 0.05, Fig. 3B). Fish caged in winter at RV had relatively little glycogen depletion, similar to summer and fall FCCL controls. Liver lesion score also varied by season and site (χ^2^ = 88.31, *P* < 0.0001), with most of the liver lesions occurring in summer and fall at RV (Fig. 3C). Among the caged fish, by far the healthiest livers came from fish deployed to RV in winter (Fig. 3C; Table 3). The most common liver lesion at RV in summer and fall was cytoplasmic inclusion bodies, an indicator of moderate starvation in Delta Smelt (Table 3, Supplementary Fig. S5, Hammock *et al.,* 2020).

Within the fall*,* glycogen depletion score varied by site (χ^2^ = 64.54, *P* < 0.0001; Fig. 4B). Nearly 100% of the caged fish in fall had severe glycogen depletion and were more depleted compared to the FCCL control fish (Steel-Dwass, *P* < 0.05; Fig. 4B). Liver lesion score also varied by site during fall (χ^2^ = 41.21, *P* < 0.0001), with elevated lesions in the caged fish (Fig. 4C). Cytoplasmic inclusion bodies were again the most common lesion in the caged fish (Supplementary Fig. S5, Hammock *et al.,* 2020; Table 3). In comparison to the livers, the gills were in relatively good condition in fall, but gill lesion score did vary (χ^2^ = 15.78, *P* = 0.0013), with fish gills at RV and YB in worse condition than the FCCL fish (Fig. 4D). The most prevalent lesion at RV during fall was ionocyte hyperplasia, followed by mucous cell hyperplasia. At YB, the most prevalent gill lesion was epithelial necrosis (Supplementary Fig. S6).

### Temperature tolerance

All fish survived the 4-h recovery period in the fall experiments with mean CTmax at 25.6 ± 1.3°C and 26.5 ± 0.9°C, of fish at RV and SM, respectively (*t* = −1.914, *P* = 0.074) (Supplementary Figs 6 and  S7).

**Figure 6:**
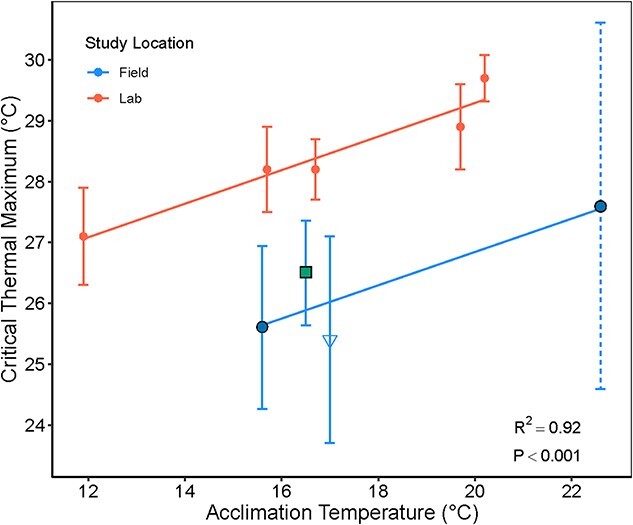
Critical thermal maximum (mean CTmax in ^0^C ± SD) of Delta Smelt by acclimation temperature in the field (blue) or laboratory (red). Field-acclimatized CTmax data are from the current study’s hatchery-reared smelt deployed in summer and fall at RV (blue circles), SM in the fall (green square) and previously wild-caught (open triangle) but lab-tested fish (Swanson *et al.,* 2000). Laboratory-acclimated Delta Smelt were reproduced from previous data (Komoroske *et al.,* 2014; Davis *et al.,* 2019a). The acclimation-rate (slope) of Delta Smelt are represented from the linear model with varying intercepts (slope = 0.28, field = 21.34, *P* < 0.001) with 2.4°C higher CTmax for laboratory-acclimated fish. The CTmax data from RV during summer (dashed line) survived trials but did not recover and may include an overestimate.

Comparative laboratory and field CTmax are shown in Fig. 6. Delta Smelt CTmax increased with increasing acclimation temperature and was higher for laboratory fish than field fish (Adj-*R*^2^ = 0.92, *P* < 0.001, Supplementary Table S6). The acclimation rate (slope) was 0.28°C per 1°C (*t* = 5.76, *P* = 0.001), with laboratory CTmax values 2.44°C higher than CTmax of fish exposed to field conditions (*t* = 8.633, *P* < 0.001). No CTmax measures were taken at YB during the fall deployments and no deployments occurred during summer for SM; however, using the field-acclimation rate provided by the regression model (CTmax = 21.34 + 0.28 (acclimation temperature)) and the mean field temperatures of 16.5°C (YB) and 23.4°C (SM) we can estimate a CTmax of 26.0°C for fish deployed at YB during fall and 27.9°C if fish were deployed in SM during the summer.

## Discussion

Hatchery Delta Smelt in field enclosures demonstrated varied physiological responses across deployment periods and sites, indicating both spatial and temporal effects on diet, growth and health, and suggestive of differences in habitat quality for Delta Smelt in the wild. Inconsistent with our predictions, temperature tolerance was similar across field sites, however, our findings showed field-acclimatized thermal tolerance differed from—and was lower than—lab-acquired thermal tolerance. Together, the results suggest that regional and seasonal differences may be monitored using caged Delta Smelt, and using cages has the potential to evaluate the effects of managed actions such as habitat restoration or augmented flows and to assess contaminant exposure in the wild. However, we also showed that caged fish were generally in worse condition in the field compared to the FCCL fish in summer and fall, exhibited a mortality event driven by high field temperature, and had signs of nutritional stress, perhaps because the cages prevented natural foraging or refuge-seeking behaviour. Additionally, we encountered biofouling of cages, particularly in summer and fall. Thus, as with any field experiment, results must be interpreted cautiously, and alternative experimental designs (e.g. ‘Before-After-Control Index, BACI) and biofouling-control methods (e.g. cage material, transfer to clean cages) should be considered in testing future aspects of fish physiological responses.

### Survival and condition

By caging Delta Smelt across three seasons and throughout the recently observed range of wild Delta Smelt, we showed that habitat conditions influenced survival and health. For example, caged fish at RV on the Sacramento River demonstrated a strong seasonal trend with 100% survival in the winter–spring, but with survival decreasing in the summer and fall by 33 and 11%, respectively (Table 1). Enclosure location also presented trends in survival but was relatively dependent on the season, such that in cooler months survival was similar at both the terminal SDWSC and RV, but in warmer months, survival differed by as much as 100%. Low survival in the summer is consistent with studies regarding temperature and food limitation (Davis *et al.,* 2022; Smith and Nobriga, 2023), with one study showing similar mass-mortality of hatchery-reared Delta Smelt in early summer when exposed to seminatural field temperatures of 27°C (Hung *et al.,* 2019). Studies of Delta Smelt have indicated that juvenile and subadult Delta Smelt are sensitive to chronic food limitation during the summer and fall, making this life stage a ‘pinch point’ for the species (Bennett, 2005; Maunder and Deriso, 2011; Moyle *et al.,* 2016; Hammock *et al.,* 2021; Smith and Nobriga, 2023).

After the four-week deployments in the wild, changes in growth of Delta Smelt were consistent with the survival results. Only fish in the winter at RV experienced positive growth and increased condition factor, suggesting conditions in the field during the winter were better for Delta Smelt than even the culture facility or later that winter at the SDWSC (Supplementary Fig. S1). In contrast, fish in the fall experienced the greatest decrease in weight and condition factor suggesting that conditions in the fall may be more limiting than in the winter for wild Delta Smelt. Inconsistent survival and growth results in the fall compared to summer; however, indicate physiology is also influenced by location with different habitat conditions. For example, RV smelt survived better in the fall compared to summer (Table 1), likely driven by lower fall temperatures, but summer fish had less negative changes in condition and weight compared to fall (Fig. 3A). This finding may be a result of several factors such as age of fish and/or habitat quality. For example, summer fish were somewhat younger (by ~70 days) than the fall fish, which may have led to differences in direction of energy, with younger fish having greater growth rates than older fish that may start to become sexually mature (LaCava *et al.,* 2023). Summer and fallfish were also tagged differently (adipose fin clipped vs. VIA tag), although significant impacts to growth or survival are not expected from different tagging methods (Sandford *et al.,* 2020). In addition to age, differences in water temperature between seasons likely influenced growth. For example, adult Delta Smelt would have lower growth rates than juveniles but the higher temperatures in the summer may have offset that ontogenetic effect. Mean summer temperatures were 22.7°C compared to a temperature in the fall of 15.6°C at RV, and warmer temperatures (without extreme temperature stress) can increase growth given sufficient prey as demonstrated in enclosure studies of juvenile Coho Salmon (Lusardi *et al.,* 2020) and Chinook Salmon deployed across different riverine and floodplain conditions (Jeffres *et al.,* 2008). Within the fall season, all sites maintained similar changes in weight (Supplementary Fig. S2), while the YB was the only site that maintained similar condition to the FCCL control (the group with the highest condition factor, Fig. 4A). This result was surprising given previous findings of juvenile Chinook Salmon (Jeffres *et al.,* 2008; Aha *et al.,* 2021) and the Longjaw Mudsucker (*Gillichthys mirabilis*; Forrester *et al.,* 2003) that showed growth was affected by location.

Negative field effects appeared to reduce the histological condition of the livers of caged Delta Smelt for all but the RV winter deployment. For all other deployments, the caged fish exhibited an array of indicators of starvation in Delta Smelt (Hammock *et al.,* 2020) as well as other fishes (Karatas *et al.,* 2021; Elbialy *et al.,* 2022). These included liver glycogen depletion, autophagosomes and single cell necrosis in the liver. Each deployment occurred in areas with chronically low pelagic productivity, and the enclosures prevented a normally vagile fish from foraging more naturally. Moreover, the gills of the caged fish were in relatively good condition, suggesting that the livers were probably less impacted by contaminants in the water, but by food limitation and/or contaminant exposure from the diet. Results of elevated liver lesions and depleted glycogen of caged Delta Smelt across habitats are consistent with food limitation studies of Delta Smelt and recaptured hatchery released Delta Smelt findings (Dhayalan *et al.,* 2023). However, the winter RV deployment demonstrates that Delta Smelt can be held in enclosures in the Delta at least one month without ill-effects, if pelagic conditions are suitable (i.e. sufficient prey for a given temperature). Thus, in the future, enclosures could be used to assess water quality and pelagic prey availability if supplemental food is provided to a subset of enclosures and biofouling is prevented; although mobility of fish would still be limited. Determining if positive growth and glycogen restoration could occur in summer and fall by subsidizing food can help to develop zooplankton thresholds and inform bioenergetic models and efficacy of habitat and food management actions.

We were surprised that livers of caged fish from SM were in the worst condition, as demonstrated by the highest levels of liver glycogen depletion, mean lesions and single cell necrosis. Wild Delta Smelt collected from SM have historically exhibited relatively good condition (Hammock *et al.,* 2015, 2021), presumably because SM contains a considerable area of relatively productive, intact tidal wetlands (Meng *et al.,* 1994; Moyle *et al.,* 2016; Baumsteiger *et al.,* 2017; Hammock *et al.,* 2019). Since the gills of fish from SM were in excellent condition, nutritional stress is likely the cause for the poor condition of the livers. We suggest two explanations for this discrepancy. First, water temperature during fall was slightly higher in SM than the other locations, which would have increased metabolic demand and exacerbated any food limitation. Second, SM has low pelagic zooplankton biomass compared to the other regions in our study, especially considering the relatively good condition of resident Delta Smelt (Hammock *et al.,* 2021). This suggests that Delta Smelt may be especially dependent on foraging behaviour in the region, possibly eating prey that are not sampled by routine zooplankton monitoring (Hammock *et al.,* 2019). Thus, our results do not necessarily indicate that SM is poor habitat for Delta Smelt compared to other deployment locations, despite relatively low pelagic prey availability (Hammock *et al.,* 2021).

Although the gills of fish deployed in enclosures were in good condition overall, there were signs of possible contaminant exposure at YB in the fall, which was the only deployment with elevated gill necrosis. While fish deployed in RV and YB during fall showed elevated ionocyte hyperplasia, that lesion varies strongly with salinity and may therefore be a response to changing water quality from the hatchery (FCCL) to the field (Teh *et al.,* 2020). Although not analyzed in our study, chemical contaminants including heavy metals, current-use pesticides from agricultural applications and legacy organochlorine insecticides (DDT and its metabolites) have been routinely detected in YB (Smalling *et al.,* 2007; Jabusch *et al.,* 2018; De Parsia *et al.,* 2019; Orlando *et al.,* 2020), including detections in juvenile Chinook Salmon tissue and their prey (Anzalone *et al.,* 2022).

### Food availability and consumption

The zooplankton and diet analyses showed clear changes across region and season. Winter deployments were characterized primarily by cyclopoid copepods and cladocera in both diet and zooplankton samples, whereas calanoid copepods gained prominence in summer and fall in RV (Fig. 5). This aligns with the population dynamics of the dominant calanoid copepod in the freshwater reaches of the Delta, *Pseudodiaptomus forbesi* (Kimmerer *et al.,* 2017). Regionally, SM zooplankton was dominated by cyclopoid copepods in the fall, while RV and the YB had more calanoid copepods (Fig. 5). This is likely due to dominance of the cyclopoid copepod *Limnoithona tetraspina*, which is one of the most abundant zooplankton in the low salinity zone in the fall (York *et al.,* 2013) and shown in Supplementary Fig. S3. *L. tetraspina* is considered a less valuable prey item than calanoid copepods due to its small size (Bouley and Kimmerer, 2006; Slater and Baxter, 2014). While cyclopoids were the most abundant prey item in the diets of caged smelt in SM (Fig. 5), most of that was the genus *Acanthocyclops*, which was rare in the marsh zooplankton community but still selected for (Supplementary Figs. S3 and S4). Given that SM had the most severe signs of starvation of any deployment, the results may support the notion that *L. tetraspina* is not a valuable prey item.

Analyses comparing diet to zooplankton abundance found only small differences between diet and zooplankton samples across seasons and regions (Table S4). This was somewhat surprising because diet samples frequently included amphipods and diptera that were completely absent from the zooplankton samples. During the YB deployment, in particular, most of the diet contents were amphipods, whereas zooplankton samples were dominated by ostracods, copepods and cladocera (Fig. 5). The enclosures provided a substrate for biofouling and colonization by epibenthic and epiphytic organisms, including amphipods, which may explain the difference between the zooplankton tows and diet samples. These epibenthic and epiphytic organisms may be more abundant in enclosures in warmer seasons (summer, fall) when biofouling was worse. Contrastingly, similar diet and community zooplankton compositions were observed in the cooler winter season. The low *R*^2^ from the PERMANOVA may be due to the high variation in diet contents, and the low replication in zooplankton samples. Additional zooplankton samples and samples from the biofouling communities in future enclosure deployments may be needed to better characterize differences between diet and zooplankton communities.

### Thermal tolerance

Based on regression analyses, field-acclimatized hatchery Delta Smelt were roughly 2.4°C less thermally tolerant than laboratory fish (Fig. 6). Compared to static, controlled and homogenous laboratory conditions, the observed field conditions were more thermally variable (Fig. 2) due to daily changes in air temperature and tides. Delta Smelt may have accrued greater energetic costs (i.e. stress) dealing with fluctuating daily temperatures. Thermal tolerance of Delta Smelt exposed to fluctuating thermal regimes in a laboratory has not been investigated; however, energetic costs of Atlantic Salmon (*Salmo salar*) under fluctuating thermal regimes were greater than exposures to the same mean temperature in a static regime (Beauregard *et al.,* 2013). Other water quality parameters (e.g. turbidity, conductivity and dissolved oxygen) and biotic factors such as food limitation in the field may have also influenced thermal tolerance (Ern *et al.,* 2016; Lee *et al.,* 2016; Firth *et al.,* 2021) as demonstrated by moderate to severe glycogen depleted livers (Figs 3B and 4B) and decreased condition (Fig. 4A). Interactions between food limitation, glycogen and tolerance have been shown to vary by species. For example, CTmax of Green Sturgeon (*Acipenser medirostris*) decreased with food-restriction whereas White Sturgeon (*Acipenser transmontanus*) CTmax was relatively unchanged by food limitation and reduced growth (Lee *et al.,* 2016; Rodgers *et al.,* 2019) and CTmax of juvenile Coho Salmon increased in response to fluctuating warm temperature regimes independent of glycogen depletion (Corey *et al.,* 2017). The daily minimum (i.e. night-time) temperatures in the fluctuating field-acclimatization (i.e. caged Delta Smelt) may also have contributed to decreased thermal tolerance compared to laboratory-acclimated fish. Increasing minimum temperatures (with the same maximum temperatures) in a fluctuating regime has shown to increase thermal tolerance in the western mosquito fish, *Gambusia affinis* (Otto, 1974), though not for juvenile Coho Salmon (Corey *et al.,* 2017). Lastly, it is important to note that the present study findings support the initial methodology decision to acclimate the caged fish to 18°C for one week at FCCL prior to field deployments. Prior to acclimation to 18°C, all Delta Smelt were kept at 12°C following aquaculture protocols for tagging or adipose fin clipping. If fish remained at 12°C and were not warm acclimated to 18°C prior to deployments, significant mortality would likely have occurred with the summer temperatures exceeding the estimated tolerance limits of 12°C-acclimated fish at 24.7°C (calculated using the conservative field-acclimation rate equation).

We suspect that seasonal acclimatization, a common physiological mechanism in fishes, occurred in our study. Comparative results at RV showed that thermal tolerance of caged Delta Smelt in summer temperatures was greater than in the fall, though the summer fish did not survive recovery so interpretation requires caution as it may be an overestimate. During hot summer months, Delta Smelt likely re-direct energy to increasing heat-shock response mechanisms (Iwama *et al.,* 1999), whereas during fall, temperature regulation mechanisms may be reduced to conserve energy (Clarke, 1993). However, it is important to remember that while thermal energetic costs may be reduced in cooler temperatures, Delta Smelt may still have elevated physiological costs depending on conductivity, turbidity, and food availability. The drastic decrease in water temperature from summer to fall likely led to a decrease in the thermal tolerance of caged fish. RV and SM had similar fall water temperatures with a mean temperature of 15.6°C at RV and 16.5°C at SM, and not surprisingly, the caged smelt had similar CTmax measures of 25.6 and 26.5°C, respectively. We considered that higher salinity in the marsh could potentially cause fish to re-direct energy from thermoregulation to osmoregulation, decreasing their tolerance; however, CTmax remained similar. These results are consistent with previous laboratory studies demonstrating Delta Smelt’s physiological tolerance and/or acclimation capacity to higher salinities >10 to 34 PSU (Komoroske *et al.,* 2014; Kammerer *et al.,* 2016; Hammock *et al.,* 2017; Davis *et al.,* 2019a; Hung *et al.,* 2022). Based on these CTmax trials, the mean experienced temperature seems to be a decent predictor for the thermal tolerance of Delta Smelt. Lastly, while we suspect the laboratory CTmax are 2.4°C higher than the field CTmax due to acclimation to controlled versus dynamic and stochastic environmental conditions that influences differential physiological and behaviour performance, we also acknowledge that the reduced thermal tolerance in the field may be attributed to transport stress. The peak stress levels of Delta Smelt measured by cortisol can occur around 30 minutes after handling stress (Pasparakis *et al.,* 2022), suggesting the reduced field CTmax may have also been influenced by handling and transfer stress from the field to the immediate CTmax experiments that occurred about 1 hour following removal. More research may be warranted to understand the influence of stress on upper temperature tolerance.

### Conclusions and implications

We conclude that hatchery Delta Smelt can successfully survive across most seasons and regions in the Estuary for extended periods of time within enclosures, though temperatures of ~22°C were limiting. Physiological condition and survival depended on season and location, which should be considered in future decisions for cage deployments. Additional locations should be considered in future deployments across seasons including historical tidal wetland habitat (e.g. Cache Slough Complex) in the winter–spring given the species might be migrating to upstream freshwater habitat, or testing locations with aspects of temperature refuge during warmer summer–fall months since temperature appears to drive the negative effects on survival and condition. We also found that although laboratory acclimated Delta Smelt have an overall greater thermal capacity, both laboratory and field-acclimatized fish have similar acclimation rates (0.28 per 1°C). This estimated thermal acclimation rate for juvenile Delta Smelt can be a tool used to predict the thermal tolerance and survival in specific habitats based on real-time water temperature measures. Thus, to maximize survival of hatchery propagated Delta Smelt in field enclosures (and supplementation strategies) that occur during warmer seasons, fish should be warm-acclimated in the laboratory for greater acquisition of thermal tolerance. Our study highlights the need to adopt an ecological physiological approach in understanding species responses, the importance of considering seasonal and habitat interactions when conducting field studies, but also provides insights into field limitations that that may be challenging to overcome in future studies (e.g. food and behaviour limitations, biofouling). Through utilizing key ecological physiological metrics alongside cage deployments, we demonstrate the significance of these methods as important tools that can test aspects of fish physiology, growth and survival to conservation efforts for Delta Smelt.

## Supplementary Material

Web_Material_coae088

## Data Availability

The data generated for this research article vary in availability. While most data were original data generated in this research some data were from a third-party or open-data platform included in the synthesis. Continuous water quality is available on the California Data Exchange Centre (https://cdec.water.ca.gov/), survival and growth for winter enclosure deployments were reproduced from Baerwald *et al.* (2023) and available online (https://doi.org/10.6073/pasta/d526b3e0b34a28145c938fe9705e7e92), all other data is available upon request.
