# Supplementary material for: Insights from a year of field deployments inform the conservation of an endangered estuarine fish
Source: Conserv Physiol. 2024 Dec 26;12(1):coae088. doi: 10.1093/conphys/coae088 (PMC11669484; doi:10.1093/conphys/coae088)
Supplement: Web_Material_coae088 [file web_material_coae088.pdf]

SUPPLEMENTARY MATERIAL

Insights from a year of field deployments inform the conservation of an endangered estuarine fish

Table S1. Continuous water quality stations used in the study for year-round data.

**Site of Cages**	**Yolo Bypass**	**Suisun Marsh**	**Rio Vista**	**North Delta Ship Channel**
**Station Code - Name**	LIS - Lisbon Weir	BDL - Beldens Landing	RVB - Sacramento River at Rio Vista Bridge	DWS - Sacramento Deep Water Shipping Channel
**Servicing Agency**	DWR	DWR	DWR	USGS
**Link**	https://cdec.water.ca.gov/dynamicapp/staMeta?station_id=LIS	https://cdec.water.ca.gov/dynamicapp/staMeta?station_id=BDL	https://cdec.water.ca.gov/dynamicapp/staMeta?station_id=RVB	https://cdec.water.ca.gov/dynamicapp/staMeta?station_id=DWS
**Coordinates**	38.474781°, -121.588226°	38.186900°, -121.970800°	38.159737°, -121.686355°	38.256110°, -121.666670°
**Metric**	**Sensor #**	**Sensor #**	**Sensor #**	**Sensor #**
River Stage	1	1	1	1
Temperature	25	25	25	25
Conductivity	100	100	100	100
Turbidity, fnu	221	27	27	221
Chlorophyll	28	28	28	
Dissolved oxygen	61	61	61	
pH	62	62	62	

SUPPLEMENTARY MATERIAL

Table S2. Result summary growth metrics of Delta Smelt in enclosures. Winter results at Rio Vista (RV) are summarized from Baerwald *et al.* (2023). The directional change in growth (e.g., weight, length, condition) is noted based on the comparative t-test of fish before and after cage deployments; decrease, increase (and if marginal p-value) or no significant difference (ns). Sites include the Sacramento River Deep water Ship Channel (SDWSC), Suisun Marsh (SM), Yolo Bypass (YB), and the reference fish kept at the Fish Conservation and Culture Laboratory (FCCL).

**Season**	**Site**	**Fork Length**	**Weight**	**Condition Factor**
Winter	RV	Increase	Increase	Increase
Winter	SDWSC	ns	Decrease	Decrease
Summer	RV	ns	Decrease (marginal) ($t = 2.2092$, $df = 114.81$, $p = 0.029$)	Decreased ($t= 3.3645$, $df = 156.73$, $p < 0.001$)
Summer	FCCL	Increase ($t = -3.8593$, $df = 140.42$, $p = 0.0001728$)	Increase (marginal) ($t = -2.1359$, $df = 145.77$, $p = 0.034$)	Decreased ($t = 3.5696$, $df = 144.76$, $p < 0.001$)
Fall	RV	ns	Decrease ($t = 4.4429$, $df = 04.121$, $p < 0.001$)	Decrease ($t = 12.045$, $df = 106.72$, $p = 2.2e-16$)
Fall	YB	ns	Decrease ($t = 5.6128$, $df = 76.488$, $p < 0.001$)	Decrease ($t = 9.3668$, $df = 145.22$, $p < 0.001$)
Fall	SM	Decrease (marginal) ($t = 2.0638$, $df = 91.708$, $p = 0.042$)	Decrease ($t = 6.3606$, $df = 84.158$ $p < 0.001$)	Decrease ($t = 13.041$, $df = 121.67$, $p < 0.001$)
Fall	FCCL	Decrease ($t = 2.3163$, $df = 112.18$, $p = 0.022$)	Decrease ($t = 3.6275$, $df = 115.53$, $p < 0.001$)	Decrease ($t = 4.27$, $df = 142.68$, $p < 0.001$)

SUPPLEMENTARY MATERIAL

Table S3. Mixed effect model results for the effect of season (at Rio Vista) and site (within fall) on the change in condition (Delta K) and change in weight (Delta Weight). RV is Rio Vista in the Sacramento River, SM is Suisun Marsh, and YB is the Yolo Bypass.

Model	Predictor	Estimate	Std. Error	df	t value	p-value
Seasons at Rio Vista						
Delta K	Winter (int)	0.060	0.014	5.656	4.353	0.005
	Summer	-0.124	0.020	6.843	-6.056	<0.001
	Fall	-0.268	0.020	5.897	-13.594	<0.001
Delta Weight	Winter (int)	0.167	0.027	5.472	6.254	0.001
	Summer	-0.244	0.040	6.578	-6.17	<0.001
	Fall	-0.396	0.038	5.696	-10.419	<0.001
Enclosure sites within fall season						
Delta K	FCCL (int)	-0.064	0.016	9.323	-4.102	0.002
	RV	-0.086	0.021	7.642	-4.051	0.004
	SM	-0.105	0.021	7.583	-4.974	0.001
	Yolo	-0.065	0.021	7.643	-3.075	0.016
Delta Weight	FCCL (int)	-0.351	0.081	8.721	-4.342	0.002
	RV	-0.044	0.109	7.371	-0.404	0.698
	SM	-0.196	0.109	7.323	-1.786	0.115
	Yolo	-0.120	0.109	7.371	-1.094	0.308

Table S4. Results from pairwise comparisons for deltaK and deltaWeight by season (at Rio Vista) and location (sites in fall). P-values adjusted using the Bonferroni method. Sites include the Sacramento River at Rio Vista (RV), Suisun Marsh (SM), Yolo Bypass (YB), and the reference fish kept at the Fish Conservation and Culture Laboratory (FCCL).

Model	Contrast	Estimate	SE	df	t ratio	p-value
Seasons at Rio Vista						
Delta K	Winter-Summer	0.124	0.0205	6.32	6.053	0.0019
	Winter-Fall	0.268	0.0197	5.44	13.593	<.0001
	Summer-Fall	0.144	0.0207	6.56	6.964	0.0007
Delta Weight	Winter-Summer	0.244	0.0395	6.3	6.168	0.0017
	Winter-Fall	0.396	0.0381	5.46	10.418	0.0002
	Summer-Fall	0.153	0.0399	6.54	3.838	0.0173
Location within Fall						
Delta K	FCCL-RV	0.0856	0.0211	9.16	4.051	0.0167
	FCCL-SM	0.1049	0.0211	9.09	4.974	0.0045
	FCCL-YB	0.065	0.0211	9.16	3.075	0.0778
	RV-SM	0.0193	0.0199	7.29	0.968	1
	RV-YB	-0.0206	0.02	7.35	-1.031	1
	SM-YB	-0.0399	0.0199	7.29	-2.001	0.5035
Delta Weight	FCCL-RV	0.0444	0.11	8.93	0.404	1

SUPPLEMENTARY MATERIAL

	FCCL-SM	0.1957	0.11	8.87	1.786	0.6495
	FCCL-YB	0.1201	0.11	8.93	1.094	1
	RV-SM	0.1514	0.105	7.38	1.446	1
	RV-YB	0.0758	0.105	7.44	0.722	1
	SM-YB	-0.0756	0.105	7.38	-0.722	1

Table S5. Permutational MANOVA model results comparing zooplankton communities across seasons at Rio Vista, across site during the fall (i.e. Rio Vista, Suisun Marsh, Yolo Bypass), and comparing zooplankton and diet composition across seasons.

Model	Predictor	df	Sum of Sqs	R2	F-Statistic	p-value
Rio Vista Zooplankton	Season	2	4.044	0.950	192.3403	0.001
	Residual	20	0.210	0.049		
	Total	22	4.255	1		
Fall Zooplankton	Site	2	0.598	0.916	76.05657	0.001
	Residual	14	0.055	0.084		
	Total	16	0.653	1		
Rio Vista diet vs. zooplankton	Diet/Zoops	1	2.118	0.0635	15.79482	0.001
	Season	2	13.644	0.409	50.86436	0.001
	Diet/Zoops*Season	2	1.687	0.051	6.849	0.001
	Residual	129	15.884	0.4765		
	Total	134	33.333	1		
Fall diet vs. zooplankton	Site	2	14.795	0.576	98.286	0.001
	Diet/Zoops	1	1.760	0.069	23.388	0.001
	Diet/Zoops*Site	2	2.282	0.089	15.160	0.001
	Residual	91	6.849	0.267		
	Total	96	25.686	1.000		

Table S6. Results of linear model of Delta Smelt critical thermal maximum (CTmax) versus aclimation/acclimatization temperature and location (field versus lab). Residual standard error was 0.414 on 6 degrees of freedom. Adjusted R-squared was 0.9188. Overall f-statistic 46.28 on 2 and 6 degrees of freedom, $p<0.0001$.

	Estimate	Std. Error	T value	p-value
Intercept - Field	21.343	0.881	24.216	<0.0001
Acclimation Temp	0.275	0.048	5.757	0.001
Location (Lab)	2.443	0.283	8.633	0.0001

SUPPLEMENTARY MATERIAL

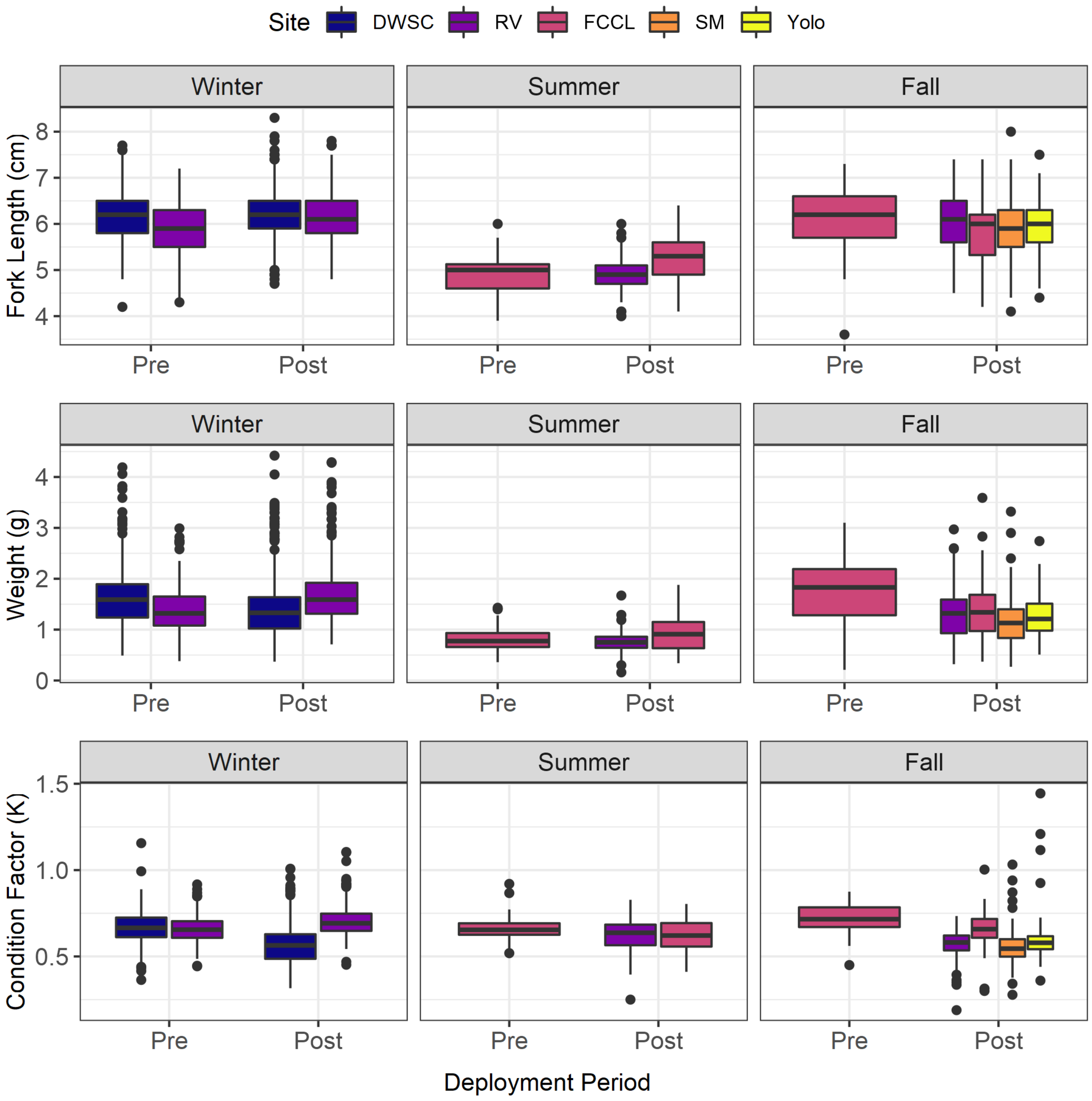

Figure S1. Length (cm), weight (g), and condition factor (K) of Delta Smelt before and after enclosure deployments across sites and season. Boxes represent the interquartile range, whiskers indicate 1.5 the interquartile range, the line is the median and points are outliers that exceed 1.5 the interquartile range. RV is Rio Vista in the Sacramento River, SDWSC is Sacramento River Deepwater Ship Channel, SM is Suisun Marsh, Yolo is the Yolo Bypass, and FCCL the reference fish kept at the Fish Conservation and Culture Lab.

SUPPLEMENTARY MATERIAL

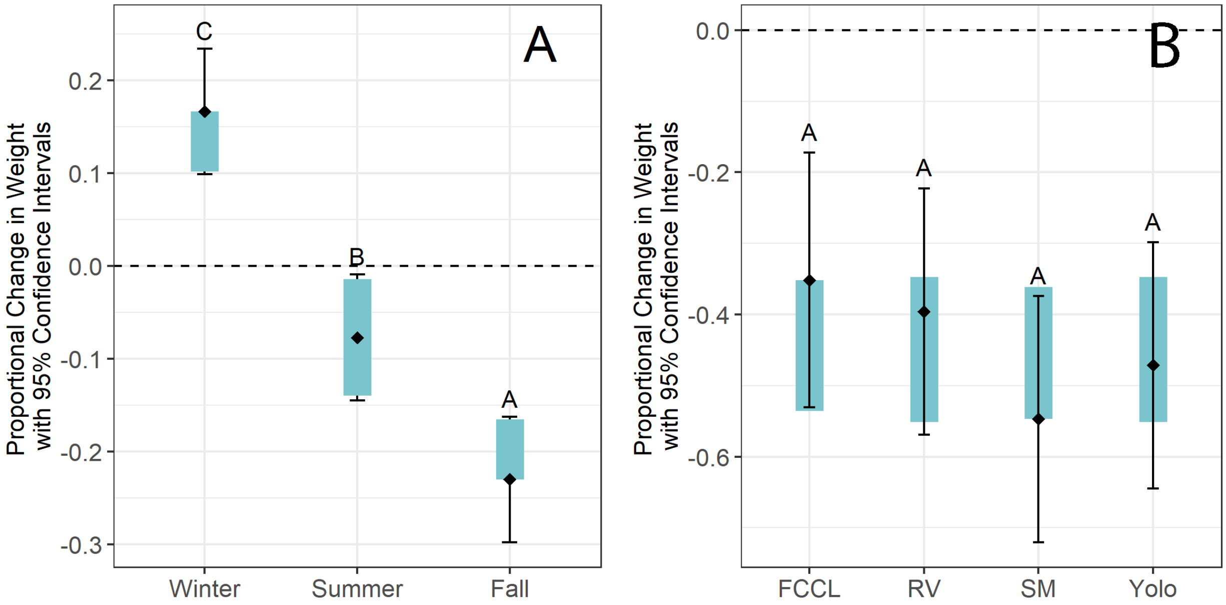

Figure S2. Modeled estimate of proportional change (with 95% confidence intervals) of weight of Delta Smelt across season (A) at Rio Vista and sites (B) in the fall. Blue bars that overlap indicate lack of significant difference. Pairwise comparisons conducted with the Bonferroni adjustment method. RV is Rio Vista in the Sacramento River, SM is Suisun Marsh, Yolo is the Yolo Bypass, and FCCL is the Fish Conservation and Culture Lab**.**

SUPPLEMENTARY MATERIAL

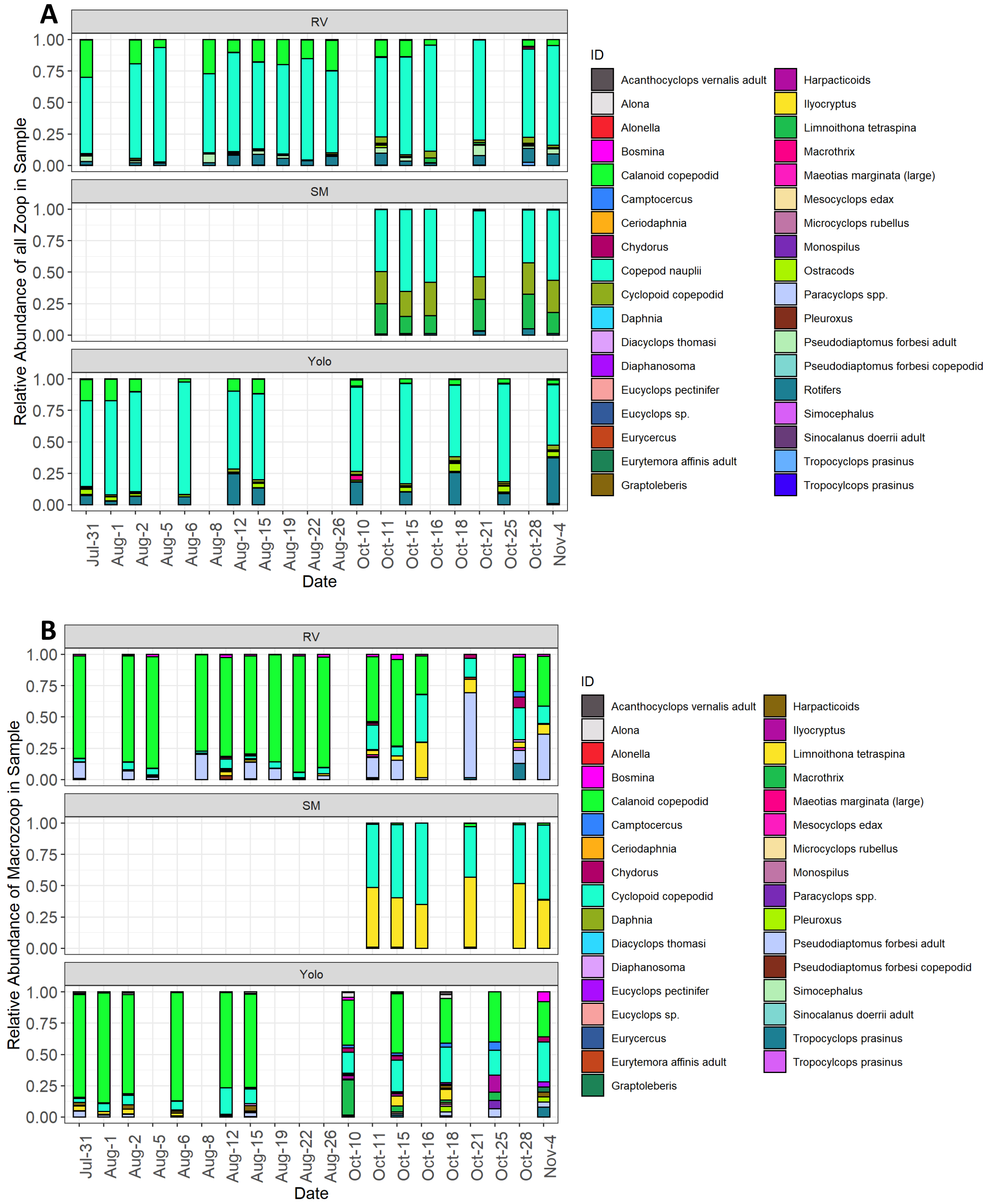

Figure S3. (A) Relative abundance of all zooplankton caught at each site during each deployment season. (B) Relative abundance of macrozooplankton caught at each site during each deployment season. RV is Rio Vista in the Sacramento River, SM is Suisun Marsh, and Yolo is the Yolo Bypass.

SUPPLEMENTARY MATERIAL

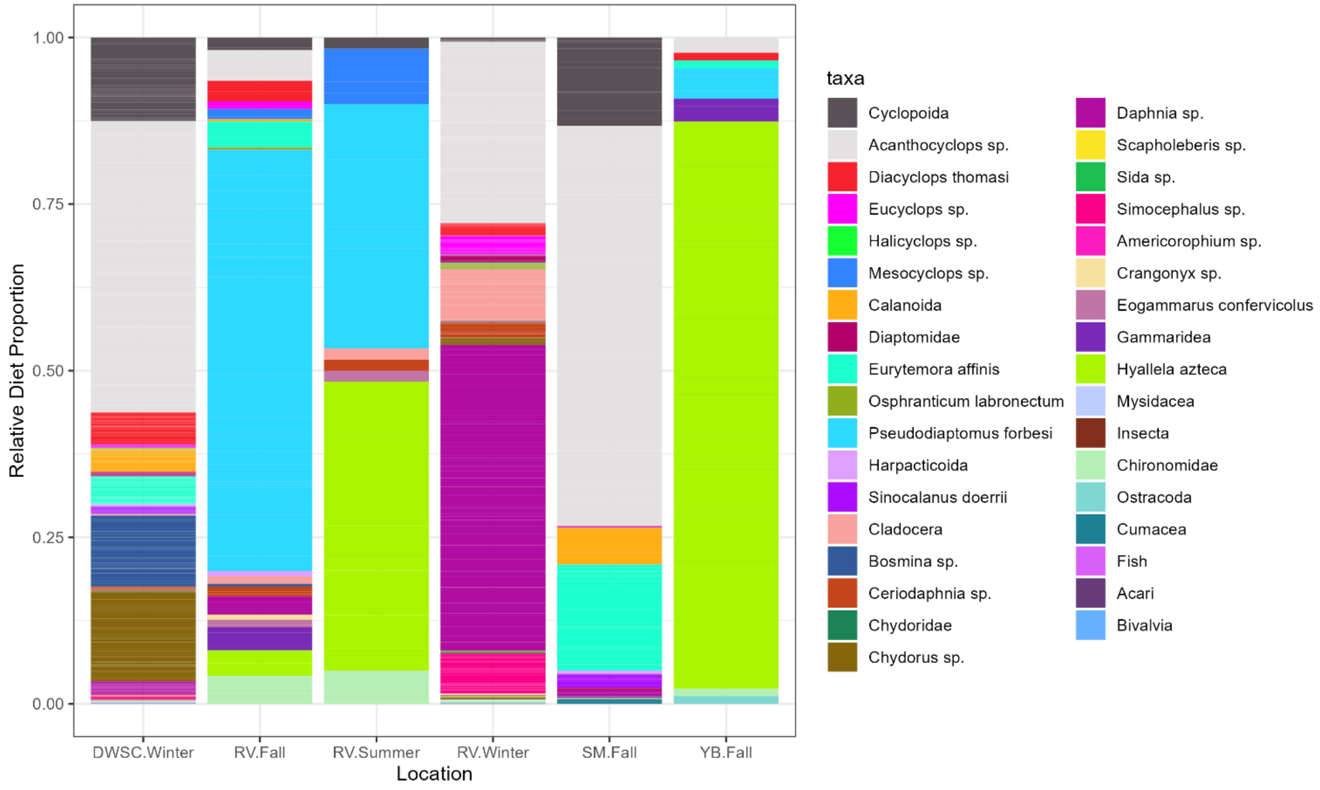

Figure S4. Contribution of different taxa to Delta Smelt diets at each location and season. SDWSC is Sacramento River Deep water Ship Channel, RV is Rio Vista in the Sacramento River, SM is Suisun Marsh, YB is the Yolo Bypass.

SUPPLEMENTARY MATERIAL

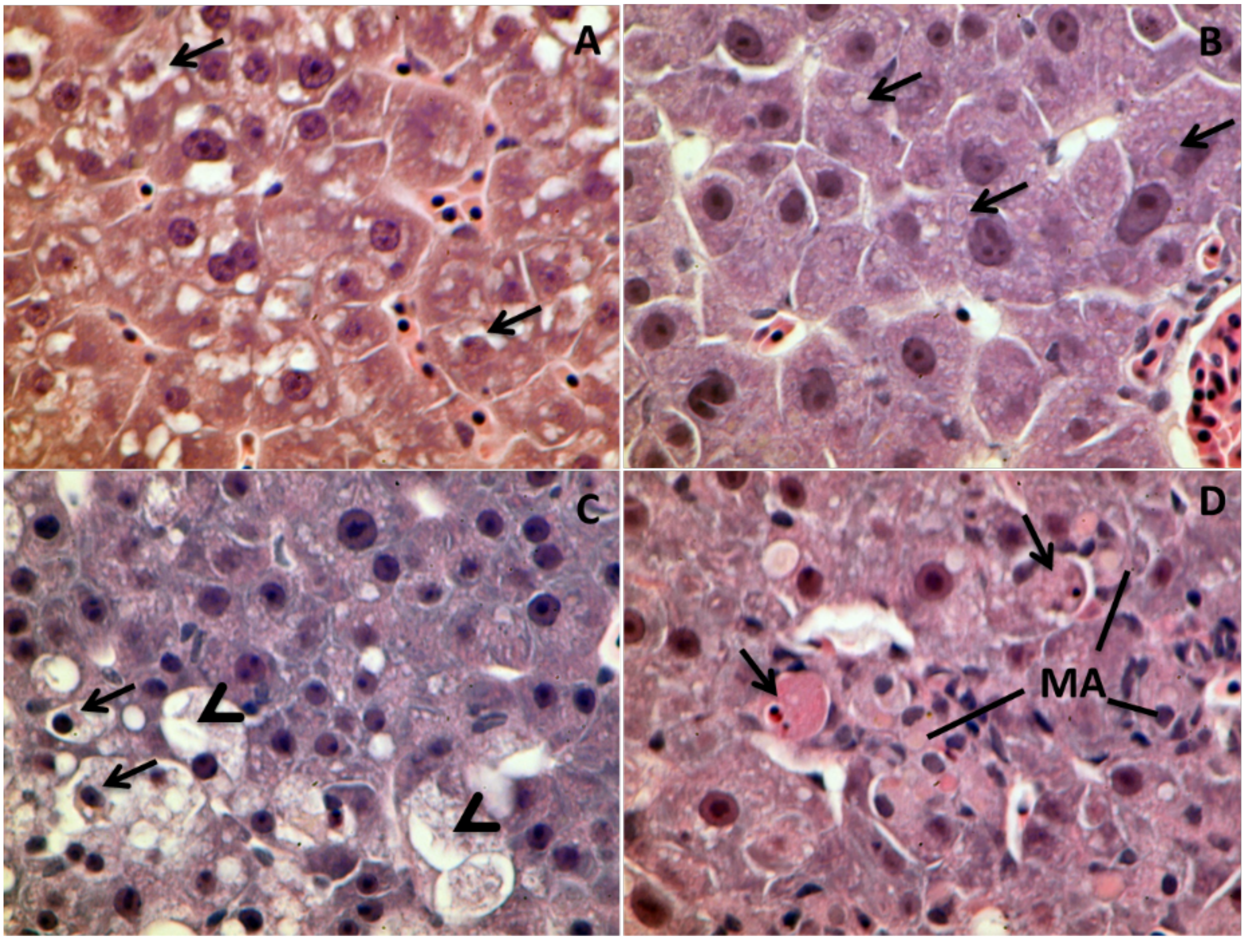

Figure S5. Livers of Delta Smelt H&E 600X. (A) Section of a glycogen-rich liver showing glycogen depots (Arrows) of FCCL control fish in fall. (B) Section of a glycogen-depleted liver of a fish caged in Yolo Bypass for 30 days. Hepatocytes are more basophilic (bluish coloration) and the nutrient stressed cells are packed with cytoplasmic inclusion bodies, presumably autophagosomes (arrows). (C) Section of a glycogen-depleted liver of a fish caged at Rio Vista during fall showing hydropic vacuolar degeneration (arrowheads) and single cell necrosis (arrows). (D) Section of a glycogen-depleted liver of a fish caged in Suisun Marsh for 30 days in fall. Note the infiltration of inflammatory cells forming a macrophage aggregate (MA) surrounding the necrotic cells (arrows).

SUPPLEMENTARY MATERIAL

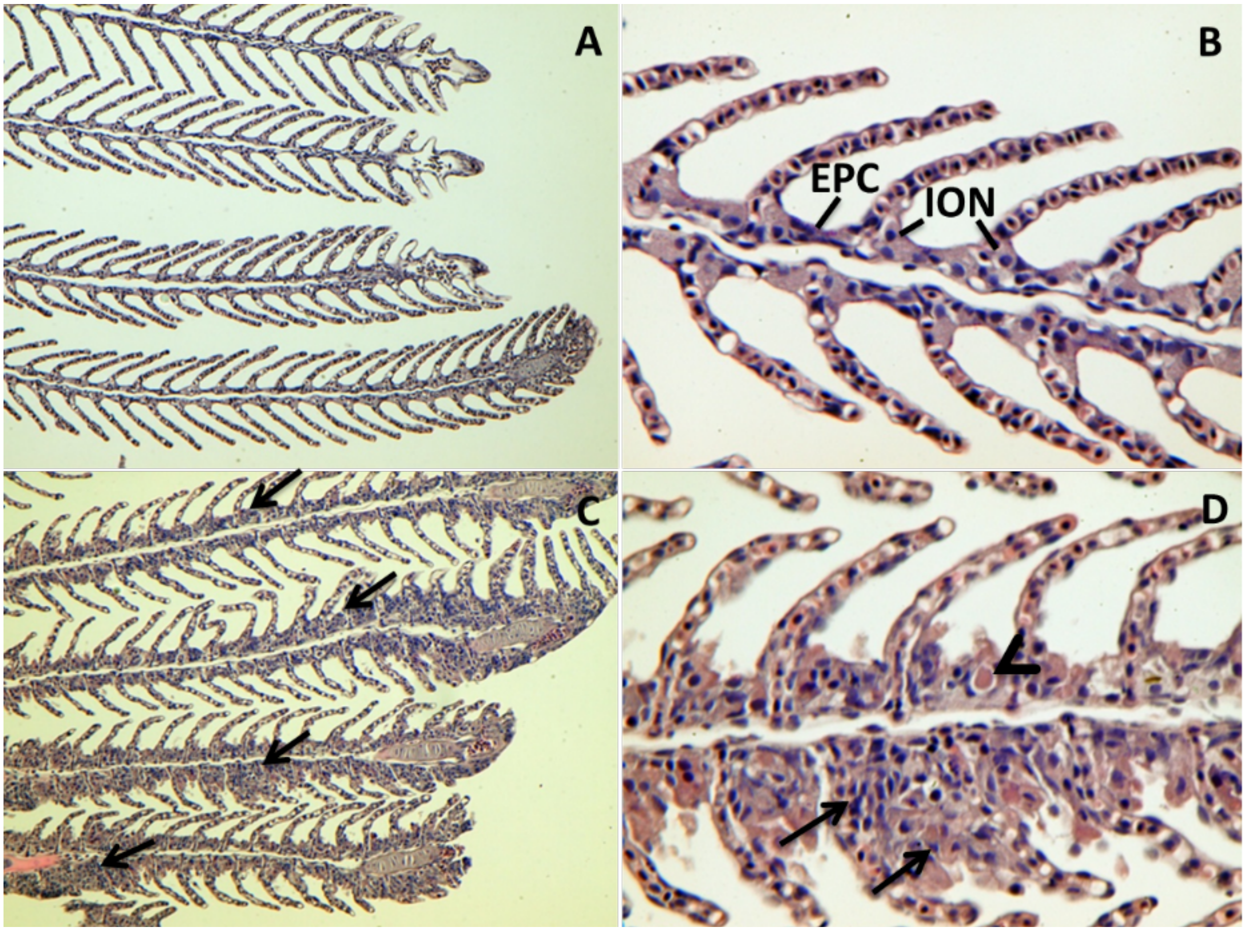

Figure S6. Gills of Delta Smelt. (A) Lower magnification showing normal comb-like architecture of gills in FCCL controls (100X). (B) higher magnification gill epithelial cells (EPC) usually 1-2 cells thick and ionocytes (ION) at junction of primary and secondary lamellae (400X). (C) Lower magnification showing epithelial cell hyperplasia (arrows) in all four primary lamella of fish caged at Yolo Bypass (100X). (D) Higher magnification of a single primary lamellae showing epithelial cell hyperplasia (arrows) and epithelial cell necrosis (arrowhead). Note epithelial layers were approximately 5-10 cells thick and have resulted in distortion and fusion of secondary lamella (400X).

SUPPLEMENTARY MATERIAL

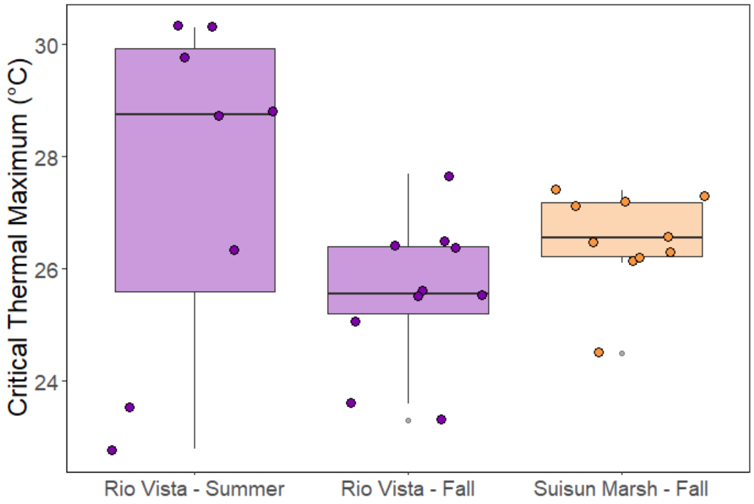

Figure S7. Critical thermal maxima (CTmax) of all fish measured in 2019 including cages in the summer at Rio Vista. The average mass (g) and fork length (cm) of measured fish were 1.0 ± 0.2 g and 5.0 ± 0.3 cm (Rio Vista- Summer), 1.4 ± 0.5 g and 6.0 ± 0.7 cm (Rio Vista- Fall), and 1.5 ± 0.6 g and 6.0 ± 0.6 cm (Suisun Marsh- Fall).
